# Geometric multidimensional representation of omic signatures

**DOI:** 10.3389/fbinf.2026.1806975

**Published:** 2026-04-17

**Authors:** Higor Almeida Cordeiro Nogueira, Enrique Medina-Acosta

**Affiliations:** Laboratório de Biotecnologia, Centro de Biociências e Biotecnologia, Universidade Estadual do Norte Fluminense, Campos dos Goytacazes, Brazil

**Keywords:** cancer metabolism, geometric representation, latent space, metabolic regulation, multi-omics, regulatory circuitries, SigPolytope

## Abstract

**Introduction:**

Multi-omic signatures are widely used in biomarker discovery, precision oncology, and systems biology, yet they are typically treated as vectors or composite scores that collapse intrinsically multidimensional biological organization into one-dimensional summaries. As a result, their internal structure, contextual dependencies, and functional coherence remain largely inaccessible.

**Methods:**

Here, we introduce a geometric framework that reconceptualizes omic signatures as multidimensional informational entities whose biological meaning arises from structural organization rather than molecular membership alone. Each signature is embedded in a shared latent space integrating regulatory, phenotypic, microenvironmental, immune, and clinical constraints, and represented as a convex polytope. This representation preserves internal organization and enables intrinsic geometric measurements—including barycenter distance, volume, anisotropy, and asymmetry—that quantify concordance, divergence, and latent complexity. We applied this framework to 24,796 metabolic regulatory circuitries reconstructed across 32 TCGA cancer types, encoded as paired regulatory and metabolic signatures in an 18-dimensional latent space.

**Results:**

Geometric analysis shows that discordance predominates: most circuitries occupy strong or extreme discordance regimes and display high-dimensional, frequently asymmetric geometries, whereas fully concordant circuitries are rare and structurally constrained. These geometric phenotypes stratify metabolic pathways and superfamilies in reproducible, non-uniform patterns that are not readily captured by conventional vector- or network-based representations.

**Discussion:**

By transforming omic signatures into measurable geometric objects, this framework provides a principled approach for the comparison and de-redundancy of multi-omic biomarkers, providing a scalable method for analyzing complex regulatory systems across cancer and beyond. All geometric representations and derived descriptors are available through the SigPolytope Shiny application (https://sigpolytope.shinyapps.io/geometricatlas/).

## 1 Introduction

### 1.1 Limits of vectorial representations in multi-omic biomarker research

The prevailing paradigm in cancer biomarker research treats omic associations as vectorial relationships linking molecular measurements to clinical or phenotypic attributes (Das et al., 2020; Romero-Garmendia and Garcia-Etxebarria, 2023; Zhang et al., 2021). Although vector representations are indispensable computational tools, using them as final biological abstractions collapses the multidimensional organization of tumor systems into one-dimensional descriptors that cannot capture coordinated biological relationships across molecular layers. Accordingly, even when termed “signatures,” these entities are often treated as lists, scores, or co-expressed gene vectors rather than as structured informational objects (Das et al., 2020; McArdle et al., 2018; Uyar et al., 2021; Ochoa and Hernandez-Lemus, 2022), despite being clinically intended to stratify patients, predict outcomes, assess risk, and guide therapeutic decisions across integrated molecular, phenotypic, and clinical dimensions (Poirion et al., 2021; Braytee et al., 2024; Kidenya and Mboowa, 2024; Wen and Wang, 2025).

**FIGURE 1:**
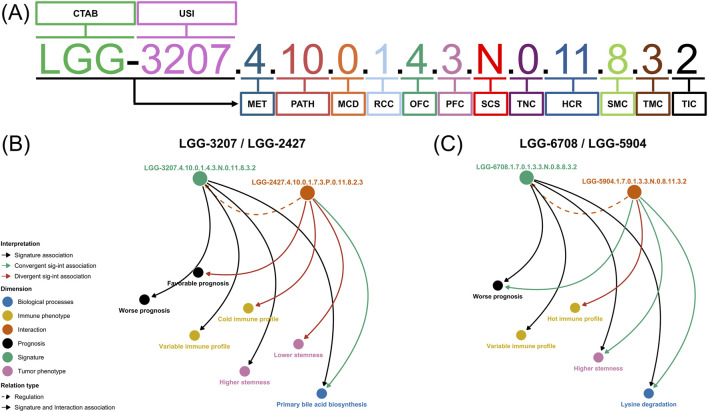
Nomenclature of multi-omic metabolic signatures and examples of regulatory circuitry configurations. **(A)** Symbolic encoding of multi-omic metabolic signatures. Panel **(A)** summarizes the nomenclature system previously introduced to encode multi-omic metabolic signatures in cancer (Almeida Cordeiro Nogueira et al., 2025). Each signature is represented by an ordered, multi-token alphanumeric identifier that integrates tumor context, metabolic class and pathway assignment, regulatory associations, omic and phenotypic layers, expression behavior, survival impact, tumor–microenvironmental context, and immune state. This encoding provides a structured annotation scheme for the regulatory circuitry datasets generated in prior work. A concise description of the nomenclature fields and encoding logic is provided in the Supplementary Material. **(B)** Divergent metabolic regulatory circuitry. Panel **(B)** illustrates a representative divergent regulatory circuitry drawn from the previously generated circuitry dataset and used here for geometric analysis. In this configuration, the paired metabolic and regulatory signatures exhibit opposing directions across one or more biological dimensions—such as phenotypic associations, survival risk profiles, or immune context—despite being linked within the same tumor type. This divergence reflects regulatory–metabolic misalignment rather than coordinated action. In the present study, such divergent circuitries are embedded into a shared latent space to quantify geometric separation and directional discordance between their component signatures. **(C)** Convergent metabolic regulatory circuitry. Panel **(C)** illustrates a representative convergent regulatory circuitry in which the paired metabolic and regulatory signatures align consistently across molecular, phenotypic, clinical, and immune dimensions. In this configuration, both components encode concordant directionality with respect to tumor–normal behavior, survival associations, and microenvironmental or immune context, reflecting coordinated regulatory control of the metabolic program within the tumor. Convergent circuitries represent cases of regulatory agreement rather than opposition and provide a conceptual contrast to divergent configurations, serving as a reference point for interpreting geometric concordance in the latent space.

To address this complexity, we previously introduced a nomenclature that explicitly maps multi-omic signatures across biological layers, mechanisms, and phenotypes (Almeida Cordeiro Nogueira et al., 2025) (Supplementary Note 1; Figure 1A). This symbolic framework supports structured interpretation through dedicated tools, including the CancerRCDShiny browser and the Multi-omic OncometabolismGPS Shiny, and enables the formal pairing of signatures into regulatory circuitries (Figure 1B).

Nevertheless, such nomenclatures and interpreter tools remain fundamentally relational (Almeida Cordeiro Nogueira et al., 2025): they indicate which components connect but do not preserve magnitude, directionality, heterogeneity, scale, or geometric structure. Consequently, two signatures with opposite survival directionality or tumor–normal behavior may appear similar when reduced to gene overlap, list-based annotations, or composite scores, despite encoding divergent biological and clinical implications. This limitation motivates representations that preserve the intrinsic multidimensional organization of omic signatures.

The geometric framework introduced here is applied, at scale, to metabolic regulatory circuitries reconstructed across multiple cancer types and described in a companion atlas manuscript (Almeida Cordeiro Nogueira et al., 2025). In the present work, these circuitries serve as an empirical substrate for evaluating the representational properties of the geometric formalism under realistic, heterogeneous, multi-omic conditions. This study is therefore not an extension of that atlas or a catalog of novel regulatory interactions, but a conceptual and methodological contribution aimed at establishing a generalizable geometric formalism for omic signatures that enables principled comparison, de-redundancy, and mechanistic interpretation beyond conventional vector- or network-based representations.

### 1.2 Signatures: from operational descriptors to structured biological entities

Although the term signature is ubiquitous in biomarker discovery, it is often used imprecisely to denote any statistical association between molecular features and phenotype. This critique does not question the clinical utility of established signatures, but the conceptual framework used to interpret their biological meaning. In this reductionist framing, signatures function as correlation-derived lists or composite scores lacking explicit regulatory architecture or structured biological organization, contextual grounding, or internal informational structure. Operationally, gene signatures are commonly defined as sets of genes whose collective expression patterns characterize biological state, subtype, prognosis, or treatment response, as exemplified by PAM50 (Parker et al., 2009), Oncotype DX (Paik et al., 2004), and the T cell–inflamed gene expression profile (Ayers et al., 2017).

While these signatures are intended to function as biological barcodes, their conceptual definition often remains loose: most aggregate molecular signals without explicitly encoding coordinated biological behavior and may not correspond to coherent regulatory programs. Consequently, signatures are often interpreted as mechanistically meaningful entities even when they remain operational descriptors with limited internal structure (McDermott et al., 2013; Cantini et al., 2018; Gygi et al., 2024). Without a more rigorous definition that frames them as multidimensional informational constructs rather than residual statistical associations, the field risks overestimating their mechanistic significance and underestimating the rigor required for clinically credible biomarker development.

An omic signature is neither a gene list, a panel, nor a simple vector, but a structured informational entity arising from coordinated molecular relationships across biological layers, regulatory hierarchies, mechanistic axes, and phenotypic contexts. It comprises integrated biomolecular components—including genes, transcripts, proteins, regulatory RNAs, and epigenomic or genomic alterations—whose organization transcends mere co-expression. By synthesizing consistent relationships across omic dimensions, biological mechanisms, and tumor phenotypes, such signatures capture the functional state of cellular processes within a defined tissue, cancer type, or clinical condition. Unlike unitary biomarkers, which encode linear associations, omic signatures represent reproducible, high-dimensional informational fields requiring a more rigorous representational framework.

### 1.3 Redefining omic signatures as emergent multi-informational entities

Building upon our prior work (Almeida Cordeiro Nogueira et al., 2025), we focus on metabolic regulatory circuitries, defined as paired entities composed of a metabolic signature and a regulatory signature (Figure 1B). Reconstructed at atlas scale, these circuitries encode regulatory alignment and opposition across molecular, phenotypic, immune, and clinical dimensions. Here, we do not re-establish their biological properties, which are reported elsewhere (Almeida Cordeiro Nogueira et al., 2025), but use them as an empirical substrate for addressing how intrinsically multidimensional paired entities should be represented, compared, and interpreted. Because they contain internal structure, directionality, and potential discordance between constituent signatures, they provide a suitable framework-development substrate.

A rigorous redefinition of omic signatures requires moving beyond the classical view of a signature as a solitary vector of measurements. That framing presumes linearity, independence among dimensions, and static interpretability—assumptions that rarely capture biological reality. Instead, omic signatures should be understood as multidimensional entities emerging from coordinated interactions across molecular, regulatory, and phenotypic layers. They integrate heterogeneous informational modalities, including discrete alterations, continuous regulatory measurements, categorical biological states, and topological attributes, whose coordinated relationships define biological meaning. This organization distinguishes signatures from traditional vectorial representations: signatures with similar gene lists may encode different regulatory architectures, whereas those with limited molecular overlap may converge toward similar biological behavior. Because such structure is context-dependent, signature organization and portability vary across tissue, lineage, microenvironment, treatment, and perturbation state. Accordingly, predictive models, evaluation frameworks, and interpretation strategies should prioritize interaction structure and cross-layer dependencies rather than marginal effects alone.

This multidimensional view naturally motivates geometric representations that preserve structure rather than collapse it. Representing signatures as convex polytopes rather than vectors provides a framework for distinguishing divergent regulatory configurations among gene-list–similar signatures and identifying functional redundancy among molecularly distinct ones. The formal geometric framework is described in the Supplementary Material.

### 1.4 Geometry as an interpretive lens for omic signatures

**FIGURE 2:**
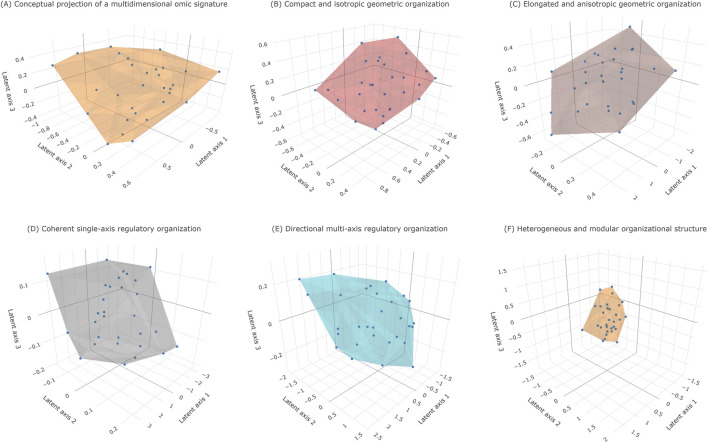
Conceptual geometric representations of omic signature organization based on simulated data. **(A)** Schematic illustration of an idealized omic signature represented as a multidimensional informational entity projected into a three-dimensional latent space. All points shown correspond to synthetically generated components (n = 30 points; convex-hull vertices = 16) and do not represent empirical molecular measurements. Points are positioned to illustrate hypothetical contributions across mechanistic, phenotypic, and contextual dimensions. The convex hull delineates the minimal geometric envelope enclosing the component cloud, providing a qualitative visualization of higher-dimensional organizational structure. **(B,C)** Conceptual examples of distinct geometric fingerprints arising from different internal organization in simulated signatures. Although the signatures contain comparable numbers of components (n = 30 points each), differences in spatial arrangement yield compact, isotropic geometries (**(B)**; hull vertices = 16) or elongated, directional geometries (**(C)**; hull vertices = 15), illustrating coherent versus axis-dominated organizational patterns, respectively. **(D,F)** Canonical geometric regimes illustrating organizational heterogeneity using synthetic point clouds. Compact and isotropic hulls (**(D)**; hull vertices = 13) indicate coherent, single-axis–dominated behavior; elongated and anisotropic hulls (**(E)**; hull vertices = 17) reflect directional structure and partial divergence; irregular and faceted hulls (**(F)**; hull vertices = 14) indicate heterogeneous organization consistent with the coexistence of multiple semi-independent biological modules. All geometries shown are generated exclusively from simulated data and are intended solely to illustrate conceptual principles of geometric organization in multidimensional omic signature space. Axis orientations, relative scales, and tick values serve as illustrative cues only and do not correspond to empirical measurements, enforced metric distances, or real omic coordinate systems. These representations do not depict experimental results but provide schematic guidance for interpreting geometric patterns in subsequent analyses of real data. (Supplementary Source Code S2 and interactive HTML versions are provided in the Supplementary Material).

Once an omic signature is conceived as a multidimensional informational entity, geometric representation becomes a natural interpretive lens because it preserves metric structure and enables quantitative comparison of organization rather than connectivity alone. In this framework, the three-dimensional convex hull serves as an accessible conceptual projection of latent structure, revealing coordination, divergence, and fragmentation among molecular components (Figure 2A; Figure 2_HTML). Figure 2 is schematic and does not depict any empirical circuitry; Panels B–F illustrate idealized organizational regimes that later recur in the analysis of real omic signatures.

Within this representation, each molecular component corresponds to a point whose position reflects its contribution across mechanistic, phenotypic, and contextual dimensions (Figure 2A). The collective distribution of these points forms a point cloud in latent space whose enclosing convex hull constitutes a geometric fingerprint of the signature. Distinct internal organizations therefore produce distinct geometric forms even when signatures share similar component counts or phenotypic associations, as illustrated by compact isotropic geometries versus elongated anisotropic geometries (Figures 2B,C).

The geometry of this envelope provides a structured representation of biological relationships encoded in the latent space. Compact isotropic forms indicate coherent organization consistent with convergence toward a dominant regulatory or phenotypic axis (Figure 2B; Figure 2D). In contrast, elongated anisotropic geometries reveal directional structure associated with axis-dominated organization, partial divergence, or hierarchical regulation across latent dimensions (Figure 2C; Figure 2E). More irregular or faceted geometries expose heterogeneous internal organization, suggesting the integration of multiple semi-independent biological modules within a single signature (Figure 2F).

Because these properties arise from structural relationships among components rather than molecular identity alone, geometry enables signatures to be compared and interpreted on the basis of organization rather than composition. In this sense, geometric representation provides an interpretive layer that complements—but does not replace—vector-based summaries or list-based annotations.

## 2 Materials and methods

### 2.1 Conceptual framework for geometric representation of omic signatures

Building upon the geometric rationale introduced above, we represent each omic signature as a finite set of biomolecular components embedded within a shared latent space (Supplementary Note 2), where each latent dimension encodes a biologically meaningful attribute, including regulatory effect, phenotypic breadth, contextual stability, or mechanistic contribution. In this framework, individual molecular components are mapped to latent coordinates that summarize their integrated multi-omic associations, enabling signatures to be treated as structured geometric objects rather than unordered molecular lists.

Within this latent space, the structural boundaries of a signature are defined by its convex envelope, corresponding to the minimal region enclosing all constituent components while preserving their multidimensional organization. This envelope provides a topology-preserving representation of internal structure and enables direct geometric interrogation of signature organization. For visualization and comparative analyses, the latent representation is projected into three dimensions while retaining the salient geometric relationships that characterize each signature. Importantly, barycenter position captures only the net directional tendency of a signature in latent space, whereas the convex hull encodes its internal geometric structure, anisotropy, and multi-axis deformation—structural properties that are invisible to distance-based analyses, clustering, or principal component analysis (PCA) coordinates alone. Throughout the manuscript, we use convex polytope to refer to the intrinsic n-dimensional geometric object defined in latent space, and convex hull to denote its minimal enclosing envelope or its three-dimensional projection used for visualization and qualitative inspection.

From this representation, we derive a family of intrinsic geometric measurements that extend beyond conventional association statistics. The volume of the convex envelope serves as a proxy for multidimensional amplitude, reflecting the breadth of biological programs engaged by the signature. The orientation of principal axes captures dominant sources of mechanistic variation, while anisotropy distinguishes signatures driven by a single coherent process from those reflecting multiple, partially orthogonal subprograms. Additional descriptors—including centroid location, spatial extent, surface morphology, and angular divergence between signatures—enable systematic comparison, classification, and stratification.

Because these measurements arise from structural organization rather than molecular identity, the framework is inherently generalizable. It is independent of any specific pathway, phenotype, or disease model and is therefore applicable beyond cancer to any biological context in which omic signatures are used to encode mechanistic hypotheses, stratification schemes, or predictive biomarkers.

By treating signatures as geometric objects rather than vectorial summaries, this approach reframes biological inference as a problem of structural quantification. Signatures can be compared, ranked, merged, or pruned on principled geometric grounds, providing a robust basis for de-redundancy, mechanistic discrimination, and interpretability. In this formulation, geometry constitutes a methodological foundation rather than a visualization accessory, rendering multi-omic signatures analytically comparable across datasets, biological contexts, and disease areas. Formal mathematical definitions and computational implementations underlying this framework are provided in the Supplementary Material.

### 2.2 Geometric representation of regulatory circuitries

To capture the multidimensional behavior of regulatory circuitries beyond conventional one-dimensional summary statistics, we embedded each circuitry into a shared latent geometric space and derived polytope-based descriptors integrating correlation structure, tumor–normal shifts, survival associations, tumor microenvironment (TME), and immune contexture.

The regulatory circuitry atlas used in this study (Almeida Cordeiro Nogueira et al., 2025) exhibits broad Pan-Cancer coverage, encompassing circuitries derived from 32 distinct TCGA cancer types, including solid tumors and hematological malignancies. These cancer types span diverse tissue origins and biological contexts, ranging from central nervous system tumors and endocrine neoplasms to gastrointestinal, gynecological, urological, and immune-related cancers. This extensive coverage ensures that the atlas provides a comprehensive foundation for the geometric analyses performed here and is not dominated by a narrow subset of tumor contexts.

Regulatory circuitries analyzed in this study were constructed using experimentally validated regulator–target relationships curated from dedicated regulatory repositories. For each metabolic or phenotypic signature (sig), the corresponding interaction component (int) was defined as the set of molecules with experimentally supported regulatory influence over one or more genes in the signature. Consequently, the sig–int pairs represent experimentally grounded regulatory circuitries rather than statistical co-associations derived solely from shared phenotypic behavior.

Each regulatory circuitry is represented as a paired entity composed of two latent profiles: one corresponding to the regulatory signature side (sig) and one to the interacting partner side (int). For each side, we constructed an 18-dimensional latent vector aggregating: (i) the Spearman rank correlation coefficient (*ρ*) between omic-layer–derived variables and their associated phenotypic attributes, together with the corresponding multiple-testing–adjusted P value; (ii) the direction of tumor-versus-normal differential expression and its −log10 Wilcoxon P value; (iii) Cox regression direction (risk versus protective) and associated P values for four survival endpoints (overall survival, disease-specific survival, disease-free interval, and progression-free interval), together with their corresponding log-rank χ^2^ statistics; and (iv) a continuous microenvironment score and a categorical immune-class assignment. Categorical variables—including tumor–normal status, survival effect (risk, protective, or non-significant), and immune class (immune-hot, cold, excluded, or intermediate)—were consistently encoded as signed numerical values (−1, 0, +1). When a survival endpoint was non-significant, the associated direction, strength, and χ^2^ contributions were explicitly set to zero to prevent non-informative axes from deforming the circuitry geometry.

Latent vectors from all circuitries (2N total vectors: N circuitries × 2 sides) were jointly embedded using PCA. Before embedding, each latent dimension was globally centered and scaled across all circuitries.

In this context, the principal axes of the geometric space are defined operationally as the leading principal components of the latent tensor. Latent axes summarize integrated variance patterns emerging from regulatory, phenotypic, immune, and clinical dimensions, without corresponding to single predefined biological variables. These axes correspond to the dominant sources of variance across the 18-dimensional representation, integrating correlation structure, tumor–normal directionality, survival-associated behavior, microenvironmental gradients, and immune states. By construction, principal components capture orthogonal directions of maximal variance in the joint latent space, providing a data-driven basis for identifying the major phenotypic and regulatory dimensions shaping circuitry geometry.

Dimensions with zero variance were retained but rescaled to unit variance to avoid numerical degeneracies while contributing no effective variance. The first three principal components defined a common three-dimensional latent coordinate system shared by all circuitries and by both sig and int profiles.

Within this three-dimensional PCA space, each circuitry was represented by a pair of local polytopes, one for the *sig* side and one for the *int* side. Distances reported throughout the manuscript correspond to Euclidean distances measured in this shared PCA coordinate system. For each 18-dimensional latent vector, we generated 36 vertices by symmetrically perturbing each latent coordinate around its barycenter in an axis-aligned manner. Perturbation magnitudes were proportional to the absolute value of each coordinate, with a minimal ε safeguard to ensure numerical stability. These vertices were projected into the PCA space and used to compute a convex hull for each side, yielding two polytopes per circuitry.

Polytope vertices are generated deterministically by applying symmetric perturbations around the latent coordinate along each axis of the representation space. These perturbations define a geometric expansion used to characterize how strongly and how broadly each circuitry engages the phenotypic dimensions encoded in the latent representation. Consequently, convex-hull properties such as volume, anisotropy, and asymmetry summarize structural features of the latent regulatory configuration rather than empirical dispersion of molecular measurements.

In this construction, internal organization reflects the multidimensional contribution structure of the latent axes, capturing how regulatory signals distribute across phenotypic dimensions encoded in the latent space. Convex-hull construction and all associated geometric measurements (including volume, anisotropy, and asymmetry) are performed in the full 18-dimensional latent space; three-dimensional convex hulls represent faithful projections of this structure used exclusively for visualization and qualitative inspection.

From this construction, we derived two complementary families of geometric descriptors. First, the barycenter distance between the sig and int polytopes was computed as the Euclidean distance between their three-dimensional centroids, providing a multidimensional measure of regulatory concordance or discordance. Small distances indicate close alignment between regulatory and interacting signatures, whereas larger distances reflect increasing phenotypic and mechanistic divergence across tumor–normal behavior, survival endpoints, microenvironmental features, and immune axes. Second, the volumes of the sig and int convex hulls were quantified as proxies for latent geometric complexity, with small volumes corresponding to low-dimensional, weakly deformed latent profiles and larger volumes indicating high-dimensional, multi-axis deformation consistent with rich, phenotype-coupled circuitry behavior.

To summarize circuitry geometry at scale, we computed for each circuitry: (i) the barycenter distance between sig and int, (ii) the convex hull volume of the sig polytope, (iii) the convex hull volume of the int polytope, and (iv) a volume asymmetry ratio comparing both sides. Using heuristic distance thresholds and data-driven volume quantiles, circuitries were assigned to categorical regimes of distance implication (high concordance, moderate discordance, strong discordance, and extreme discordance) and volume implication (low-dimensional/flat, intermediate, or high-complexity geometries, further stratified by symmetric or asymmetric volume distributions). Together, these descriptors define a geometric phenotype for each circuitry, distinguishing concordant and discordant regulatory behavior, simple versus complex latent organization, and symmetric versus asymmetric regulatory architectures across the multi-omic, immune, and survival dimensions encoded in the OncoMetabolismGPS framework (Almeida Cordeiro Nogueira et al., 2025). All geometric constructions, metric computations, regime assignments, and classifications reported in this study were generated using fixed parameters and deterministic rules, without manual curation, interactive tuning, or post-hoc adjustment of thresholds or assignments.

Detailed computational implementation of the geometric circuitry framework is provided in Supplementary Note 3, while its formal mathematical definitions are described in Supplementary Notes 4, 5; full reproducible workflows are available in Supplementary Source Code S1, 2.

### 2.3 Reconstruction of latent vectors and geometric discordance metric

Dataset S1 contains regulatory signatures derived from seven molecular modalities, including bulk-RNA gene expression, transcript isoform expression, miRNA expression, CpG methylation, copy-number variation (CNV), somatic mutation, and protein expression. These signatures originate from the regulatory circuitry atlas described in the companion study (Almeida Cordeiro Nogueira et al., 2025). Importantly, each signature in the dataset is mono-omic by construction, meaning that it is defined within a single molecular modality. Consequently, the circuitry atlas contains signatures originating from multiple molecular layers rather than composite signatures combining features from several modalities simultaneously.

Within the SigPolytope framework, these mono-omic regulatory signatures are subsequently characterized using phenotypic descriptors computed from the same TCGA patient cohorts, including survival associations, immune classification, tumor–normal directionality, and microenvironment context. These descriptors define the latent phenotypic representation used to analyze the geometric organization of regulatory circuitries. The multi-omic nature of the analysis therefore arises from the simultaneous analysis of signatures originating from multiple molecular modalities within a shared geometric framework rather than from joint latent modeling of raw multi-omic matrices.

The geometric representation introduced here does not attempt to perform dimensionality reduction of raw multi-omic sample matrices. Instead, it analyzes the structural organization of regulatory circuitries derived from those data. In this framework, the analytical objects are regulatory circuitries represented by latent phenotypic vectors summarizing pathway-level behavior across patient cohorts rather than individual tumor samples. PCA therefore serves only to define a shared coordinate system for embedding these circuitry-level latent vectors, enabling geometric comparison of regulatory structures while preserving the pathway-level abstraction of the underlying regulatory atlas.

To enable validation analyses, the latent representations used for geometric discordance calculations were reconstructed directly from the variables provided in Dataset S1. The dataset encodes the 18-dimensional latent vectors describing regulatory circuitries for both the signature and interaction components of each metabolic pathway.

For each pathway, two vectors were reconstructed representing the coordinates of the signature and interaction components in the latent space. These vectors were embedded into a shared low-dimensional representation using PCA applied to the combined set of signature and interaction latent variables. Before PCA, the 18-dimensional latent vectors were globally centered and scaled across all circuitries by subtracting the column-wise mean and dividing by the column-wise standard deviation of each latent dimension. PCA was then computed on this standardized matrix, ensuring that heterogeneous variables contribute comparably to the embedding and that geometric distances reflect covariance structure among standardized latent features rather than differences in raw variable scale. The geometric discordance metric (*dbary*) was then computed as the Euclidean distance between the barycenters of the signature and interaction vectors in the PC1–PC3 embedding space. This metric quantifies the geometric separation between the two regulatory representations within the shared latent coordinate system.

To verify the reproducibility of the geometric computation, barycenter distances recomputed from the reconstructed latent vectors were compared with the *dbary* values reported in Dataset S1 using Spearman rank correlation.

All steps of the latent vector reconstruction and barycenter distance recomputation are implemented in the supplementary validation script (Supplementary Source Code S1), available in the project repository (https://github.com/BioCancerInformatics/SigPolytope).

### 2.4 Benchmarking against baseline discordance metrics

To evaluate whether the geometric discordance metric captures information beyond conventional vector-space discordance measures, we performed benchmarking analyses comparing *dbary* with several baseline discordance measures derived from the same reconstructed latent vectors.

The following baseline metrics were computed:

- Euclidean distance between signature and interaction vectors in the reconstructed 18-dimensional latent space.
- Cosine distance between signature and interaction vectors.
- Survival-direction mismatch counts across four survival endpoints (OS, DSS, DFI, and PFI).
- Immune classification mismatch between signature and interaction components.
- Tumor–normal direction mismatch.
- Absolute differences in microenvironment score

These baseline statistics represent simpler vector-space measures of discordance derived from the same latent variables and therefore provide a direct benchmark for evaluating whether the geometric discordance metric reflects information not captured by conventional distance or mismatch statistics.

To quantify relationships between the geometric and baseline metrics, a Spearman correlation matrix was computed across all discordance measures. The resulting correlation structure was visualized as a heatmap to summarize the degree of association between the geometric representation and alternative vector-based discordance statistics.

The full benchmarking pipeline, including computation of baseline metrics and generation of the correlation heatmap, is implemented in the supplementary validation script (Supplementary Source Code S1), available in the project repository (https://github.com/BioCancerInformatics/SigPolytope).

All analyses were performed deterministically using the values present in Dataset S1 without simulation, imputation, or generation of additional variables.

### 2.5 Statistical evaluation of discordance regimes

To evaluate whether the observed concordance–discordance regime structure could arise from random pairing between molecular signatures and regulatory interaction components, we implemented a permutation-based null model. Interaction embeddings were randomly reassigned within cancer-type strata in order to preserve cohort composition while disrupting the pairing between signature and interaction components.

For each permutation replicate (B = 500), the shared PCA embedding was recomputed, barycenter distances (*dbary*) were recalculated, and circuitries were reassigned to discordance regimes using the same operational thresholds applied in the main analysis.

This procedure generated null distributions for several global geometric statistics, including the mean barycenter-distance metric and the proportions of circuitries classified as extreme discordance and high concordance. Empirical p-values were obtained by comparing the observed statistics with their permutation-based null distributions. To avoid zero-probability artifacts inherent to finite permutation samples, p-values were estimated using the bias-corrected permutation estimator $p=\left(k+1\right)\;/\;\left(B+1\right)$ , where *k* denotes the number of permutation replicates yielding a statistic greater than or equal to the observed value. and *B* denotes the total number of permutations.

### 2.6 Interactive visualization of geometric metabolic regulatory circuitries

To enable structured exploration and interpretation of the geometric results generated in this study, we developed the SigPolytope Shiny application (URL: https://sigpolytope.shinyapps.io/geometricatlas/). The application provides access to the 18-dimensional latent representations of regulatory circuitries and their corresponding three-dimensional projections, together with derived geometric descriptors including barycenter distance, convex-hull volumes, and associated implication regimes.

Circuitries can be filtered by cancer type, omic and phenotypic layers, metabolic class, pathway annotation, and metabolic cell-death processes. Individual signature–interaction pairs are visualized as interactive dual three-dimensional polytopes within a shared latent coordinate system. In parallel, the interface generates structured textual summaries derived from the standardized signature nomenclature, explicitly linking geometric properties to biological, clinical, microenvironmental, and immune features encoded in the OncoMetabolismGPS framework.

The Shiny application functions as a reproducible visualization and interrogation layer within the analytical workflow, enabling systematic examination of the geometric organization of regulatory circuitries without altering the underlying computational results. All geometric metrics reported in the Results are deterministically derived from the latent tensors and are reproducible from Dataset S1 using the provided codebase, without manual intervention or parameter tuning.

### 2.7 Conceptual definitions

To ensure conceptual clarity and terminological consistency, we introduce here the core geometric constructs that underlie the SigPolytope framework. These definitions formalize how omic signatures and regulatory interactions are represented, compared, and interpreted in latent space, and they establish a common vocabulary for the geometric metrics reported throughout the manuscript. The key terms and measures used in all subsequent analyses are summarized in Box 1.

Box 1 **Geometric concepts and definitions used in Sig Polytope.**

**Latent space**
  A shared multidimensional coordinate system derived from the integration of omic, phenotypic, immune, and clinical features. Each latent axis represents a composite dimension capturing correlated biological variation across these layers.
**Principal axes**
  The orthogonal dimensions of the latent embedding that capture dominant sources of variance across integrated omic, phenotypic, immune, and clinical features.
**Anisotropy**
  A geometric property of the convex hull describing unequal extension of a signature or circuitry across latent axes, reflecting directional dominance rather than positional variance.
**Signature (sig) and interaction (int) components**
  For each regulatory circuitry, the sig component represents the upstream regulatory entity (e.g., an ncRNA-based signature), whereas the int component represents the downstream metabolic or molecular interaction target. Each component is represented as a local polytope embedded in latent space.
**Barycenter**
  The geometric center (mean coordinate) of a polytope, summarizing the net directional contribution of all latent dimensions for a given circuitry component.
**Barycenter distance (dbary)**
  The Euclidean distance between the sig and int barycenters in the shared latent space. This metric quantifies the degree of geometric separation between the regulatory and metabolic components of a circuitry.
**Barycenter-distance regimes**
  Circuitries are discretized into four regimes based on dbary --- high concordance, moderate discordance, strong discordance, and extreme discordance. These regimes provide an interpretable categorization of continuous geometric separation and are used consistently throughout the main text and Supplementary analyses.
**Convex hull**
  The minimal geometric envelope enclosing all vertices of a polytope. Hull geometry provides a visual and quantitative summary of the internal organization of a signature or interaction component.
**Hull volume**
  A quantitative measure of the breadth of latent engagement, reflecting the number and diversity of latent dimensions contributing substantially to a given circuitry component.
**Symmetry (sig-int volume ratio)**
  The relative balance between sig and int hull volumes, indicating whether latent complexity is evenly distributed between components or dominated by one side.
**Separation-with-convergence**
  A geometric configuration in which sig and int components are spatially separated in latent space (large barycenter distance) yet exhibit concordant sign structure along specific annotated axes (e.g., survival, microenvironmental, immune). In such cases, barycenter distance captures latent-direction separation, whereas concordance reflects axis-specific functional alignment.

Formal mathematical definitions of the latent representation, convex-hull construction, and polytope-based geometric descriptors used in the SigPolytope framework are provided in the Supplementary Material (Supplementary Notes 3-5).

A schematic overview of the SigPolytope analytical workflow—from regulatory circuitry integration and latent vector construction to geometric polytope generation, descriptor extraction, and regime classification—is provided in Supplementary Figure S1.

## 3 Results

### 3.1 Geometric embedding of 24,796 regulatory circuitries in an 18-dimensional latent space

We constructed an 18-dimensional latent tensor for each signature–interaction pair and embedded all circuitries into a unified geometric representation using principal component analysis (PCA). All regulatory circuitries were embedded in a shared 18-dimensional latent space and are visualized throughout the manuscript via a common three-dimensional PCA projection derived from this space. This embedding integrates phenotypic correlation structure, tumor–normal directionality, four survival endpoints, microenvironmental scores, and immune classification into a shared latent representation. The resulting space is continuous but strongly anisotropic, indicating that regulatory circuitries occupy preferential geometric directions rather than forming a homogeneous cloud. Barycenter distances between signature and interaction components follow a long-tailed distribution, with a median of 2.59 (IQR: 1.38–3.99) and extending up to 33.2 (Dataset S1, and Dataset S1 variable descriptors in Supplementary Material), revealing the coexistence of tightly coupled circuitries and profoundly discordant regulatory–metabolic relationships.

**FIGURE 3:**
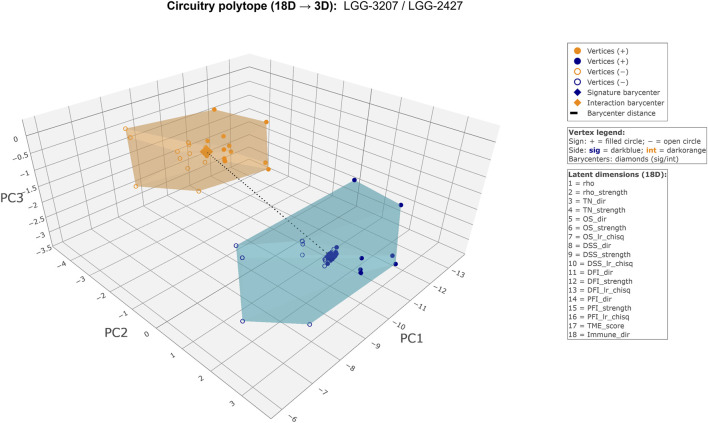
Geometric dual-polytope representation of a highly discordant metabolic regulatory circuitry. Detailed geometric exemplar of a metabolic regulatory circuitry in lower-grade glioma (LGG) linking a miRNA-based regulatory signature (sig: hsa-miR-10a-5p; miRNA expression) and a methylation-derived interaction module (int: *CYP8B1*; DNA methylation), both associated with the stemness phenotypic layer. The circuitry is embedded within lipid metabolism, specifically the primary bile acid biosynthesis pathway. Although both components map to the same phenotypic domain, they exhibit opposing biological behavior: the regulatory miRNA signature shows a strong negative association with stemness (*ρ* = −0.41), whereas the metabolic interaction module displays a positive association (*ρ* = +0.25). This opposition extends to clinical and microenvironmental dimensions, with the sig component associated with a risky survival profile, pro-tumoral microenvironment, and variable immune context, while the int component shows a protective survival association, dual microenvironmental classification, and a cold immune phenotype. Geometrically, the two components are encoded as 18-dimensional latent regulatory vectors and represented as convex dual polytopes in a shared PCA space. The large barycenter distance (7.16) places the circuitry in an extreme discordance regime, while the comparable polytope volumes (symmetric, high-complexity configuration) indicate balanced informational dispersion across layers. Together, these features define an Only Divergent circuitry, exemplifying pronounced geometric and biological dissociation between regulatory and metabolic components within a shared metabolic pathway (Supplementary Source Code S2) and corresponding interactive HTML figure hosted externally in Supplementary Material).

Figure 3 (Figure 3_HTML) provides an illustrative example of this latent organization, illustrating how extreme geometric separation corresponds to opposing multi-layer phenotypic orientations encoded in the latent representation of a single circuitry. In this example, regulatory and interaction components associated with the same phenotypic axis (stemness) localize to distant regions of the latent space, reflecting opposing correlation directionality, survival implications, and microenvironmental and immune contexts.

The large barycenter distance and symmetric, high-complexity polytope configuration shown in Figure 3 illustrate how divergent phenotypic associations are represented as spatial separation in the latent embedding, providing an interpretable geometric summary of their relative orientation in latent space.

### 3.2 Prevalence and structure of barycenter-distance regimes

Circuitries were assigned to four barycenter-distance (*dbary*) regimes using fixed thresholds in the shared three-dimensional PCA space: high concordance (*dbary* < 0.5), moderate discordance (0.5 ≤ *dbary* < 1.5), strong discordance (1.5 ≤ *dbary* < 2.5), and extreme discordance (*dbary* ≥ 2.5). These regimes discretize a continuous geometric spectrum and should be interpreted as operational bins rather than intrinsic biological states. This classification summarizes the magnitude of geometric separation between the signature and interaction components of each circuitry in latent space (Table 1). Importantly, geometric separation does not necessarily imply functional opposition, as spatial divergence in latent space may coexist with concordant behavior along specific annotated biological axes. Because barycenters encode the net directional contribution of all latent dimensions, increasing barycenter distance reflects progressively stronger divergence in overall latent orientation between the regulatory and metabolic sides of a circuitry.

**TABLE 1:** Distribution of regulatory circuitries across barycenter-distance and volume-based geometric regimes.

(A) Barycenter-distance regimes		
Barycenter-distance implication regime	Count (n)	Percentage (%)
Extreme discordance	12869	51.9
Moderate discordance	5360	21.6
Strong discordance	5190	20.9
High concordance	1377	5.6

(B) Geometric volume-implication regimes		
Geometric volume implication regime	Count (n)	Percentage (%)
High-complexity multidimensional, strongly asymmetric geometry	6463	26.1
Low-dimensional, strongly asymmetric geometry	5309	21.4
Intermediate-complexity, strongly asymmetric geometry	3830	15.4
Intermediate-complexity, moderately asymmetric geometry	2836	11.4
Low-dimensional, symmetric geometry	1979	8
Intermediate-complexity, symmetric geometry	1601	6.5
High-complexity multidimensional, moderately asymmetric geometry	1031	4.2
Low-dimensional, moderately asymmetric geometry	978	3.9
High-complexity multidimensional, symmetric geometry	769	3.1

The distribution of circuitries across barycenter-distance regimes was dominated by discordant geometric classes. Extreme discordance represented the largest regime, accounting for more than half of all circuitries, whereas strong discordance formed a second substantial cluster. Moderate discordance contributed an additional broad intermediate region. High concordance was comparatively rare, comprising only a small fraction of the atlas. These results demonstrate that most circuitries encode divergent latent behaviors in at least one of the major phenotypic axes. Table 1 provides the exact frequencies for all four classes. This pattern establishes discordance as the principal organizing feature of the regulatory landscape, a characteristic readily visible in the separation of barycenters shown in Figure 3.

### 3.3 Convex-hull geometry defines nine classes of latent complexity and asymmetry

Local multidimensional structure around each circuitry was resolved using convex hulls constructed from barycenter-centered perturbations along all 18 latent axes. Hull volume served as a proxy for latent complexity, allowing classification into low-dimensional, intermediate-complexity, and high-complexity geometries. The ratio of signature-to-interaction volumes quantified geometric asymmetry, leading to three possible symmetry states. Together, these measures defined nine mutually exclusive geometric classes.

Across the full Dataset S1, high-complexity multidimensional geometries were prominent, particularly when combined with strong asymmetry between signature and interaction sides. This combination emerged as the single most frequent class, emphasizing that many circuitries engage multiple latent axes yet distribute this complexity unevenly across their two components. Low-dimensional flat geometries were observed but constituted a minority, and the symmetric subset within this tier represented only a modest proportion of the atlas. Intermediate-complexity geometries spanned both symmetric and asymmetric classes and occupied a transitional position in the geometric hierarchy. Table 1 summarizes the distribution of circuitries across all nine structural categories. Supplementary Figure S2 (Figure S2_HTML) presents representative convex-hull geometries arranged by latent complexity tier (low, intermediate, high) and symmetry state (symmetric vs. signature-dominant), providing a visual atlas of the major observed geometric classes.

### 3.4 Joint patterns of discordance and latent complexity define four principal geometric phenotypes

The combination of barycenter-distance regimes with the nine convex-hull categories produced four recurrent geometric phenotypes. Concordant and low-dimensional circuitries formed compact, symmetric dual polytopes with minimal axis deformation. This group was the rarest but represented the most coherent regulatory units in Dataset S1. Concordant but high-dimensional circuitries also emerged, forming large symmetric hulls in which multiple latent axes contributed coordinated deformation on both sides; these circuitries exhibited substantial internal richness while maintaining minimal divergence between their signature and interaction components.

In contrast, discordant low-dimensional circuitries showed pronounced barycenter separation despite minimal hull inflation, indicating sharply opposed yet geometrically simple regulatory programs. The most prevalent phenotype combined discordance with high dimensionality and strong asymmetry. These circuitries displayed large, irregular, and often elongated polytopes with clear separation between signature and interaction barycenters. This dominant class reflects complex and imbalanced phenotypic influences, frequently involving divergent contributions across survival, immune, tumor–normal, and microenvironmental axes.

Supplementary Figure S3 (Figure S3_HTML) provides population-level exemplars of these four geometric phenotypes. Panel A illustrates a discordant yet low-dimensional asymmetric circuitry, characterized by marked barycenter separation with limited internal complexity. Panel B shows a highly concordant, symmetric, and low-dimensional circuitry, representing compact and internally coherent regulatory organization. Panel C exemplifies extreme discordance with intermediate internal complexity, highlighting phenotypic convergence accompanied by immune and survival divergence. Panel D captures the extreme end of the spectrum, combining maximal discordance with high-dimensional and strongly asymmetric geometry, reflecting deeply imbalanced regulatory and phenotypic influences. Together, these exemplars illustrate how the four principal geometric phenotypes manifest across cancer types and metabolic contexts at the population level.

### 3.5 Representative circuitries illustrate distinct geometric behaviors

**FIGURE 4:**
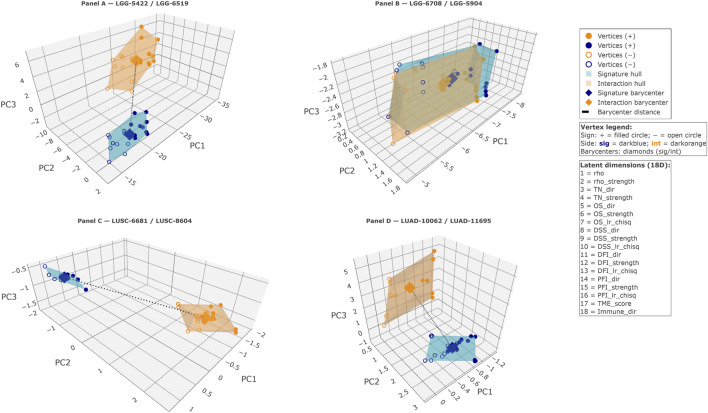
Canonical, visually interpretable archetypes of the joint distance–volume implication space. Panels **(A–D)** are ordered to reflect increasing biological resolution rather than increasing geometric distance. **(A)** Dual-polytope representation of a metabolic regulatory circuitry in lower-grade glioma (LGG) linking a miRNA-based regulatory signature (sig: hsa-miR-615-3p; miRNA expression; stemness) and a CNV-derived interaction module (int: PSAP; CNV; stemness), anchored to lipid metabolism, specifically the sphingolipid metabolism pathway. The two components are widely separated in latent space (barycenter distance = 13.67), defining an extreme discordance regime with high complexity, moderately asymmetric hull geometry. Biologically, the sig component is risky, pro-tumoral, and immune-variable, whereas the int component is protective, associated with a dual microenvironment and a cold immune state, together defining an Only Divergent circuitry. **(B)** A dual-polytope embedding of a metabolic regulatory circuitry in LGG, coupling a regulatory signature (sig) and an interaction module (int) that are highly aligned across molecular and phenotypic dimensions. The regulatory component corresponds to the lncRNA *SNHG12*, derived from CNV and associated with stemness, while the interaction component corresponds to *KMT2B*, likewise CNV-derived and mapped to the stemness phenotypic layer. This circuitry is situated within amino acid metabolism, specifically the lysine degradation pathway. In the shared latent space, the two components localize in close proximity (barycenter distance = 0.20), consistent with a high-concordance geometric regime and a high-complexity, symmetric hull configuration. Biologically, both sig and int components display meaningful risky survival associations and a pro-tumoral microenvironment, while differing at the immune level (variable versus hot), yielding a circuitry characterized by phenotypic and survival convergence with immune divergence. **(C)** A dual-polytope representation of a metabolic regulatory circuitry in LUSC contrasting a miRNA-based regulatory signature (sig) with a transcriptionally defined interaction module (int) that diverge despite shared phenotypic grounding. The regulatory component corresponds to hsa-miR-30b-5p, derived from miRNA expression and associated with stemness, whereas the interaction component corresponds to *LPCAT1*, derived from gene expression and likewise mapped to the stemness phenotypic layer. This circuitry is embedded within lipid metabolism, specifically the ether lipid metabolism pathway. In latent space, the two components occupy clearly separated regions (barycenter distance = 3.63), consistent with an extreme discordance regime and an intermediate-complexity, strongly asymmetric hull configuration dominated by the interaction component. Biologically, the sig module displays a meaningful protective survival association together with a pro-tumoral microenvironment and variable immune context, whereas the int module is meaningfully risky, associated with an anti-tumoral microenvironment and a hot immune state, yielding a circuitry characterized by phenotypic convergence with divergent survival and immune behavior. **(D)** Dual-polytope depiction of a metabolic regulatory circuitry in LUAD juxtaposing a miRNA-based regulatory signature (sig) and a CNV-derived interaction module (int) that diverge geometrically while converging biologically. The regulatory component corresponds to hsa-miR-550a-5p, derived from miRNA expression and associated with microsatellite instability, whereas the interaction component corresponds to *NT5C1B*, derived from CNV and linked to tumor mutational burden. This circuitry is anchored to nucleotide metabolism, specifically the purine metabolism pathway, in the LUAD context. In the latent embedding, the two components occupy distant regions (barycenter distance = 4.64), consistent with an extreme discordance regime and a high-complexity, moderately asymmetric hull configuration. Biologically, both sig and int components exhibit meaningful risky survival associations, align with an anti-tumoral microenvironment, and share a cold immune phenotype, collectively defining an Only Convergent circuitry despite pronounced geometric separation. (Supplementary Source Code S2) and link to the corresponding HTML figure are in Supplementary Material).

Detailed visualization of selected circuitries revealed a set of canonical geometric archetypes occupying distinct regions of the joint distance–volume implication space (Figure 4, F4_HTML). Panel A illustrates an extreme discordance archetype in which regulatory and interaction components are widely separated in latent space and form high-complexity, moderately asymmetric dual polytopes, corresponding to pronounced functional divergence between the two sides of the circuitry. By contrast, Panel B exemplifies a high-concordance regime, where regulatory and interaction components localize in close proximity and form symmetric, high-complexity hulls, reflecting coordinated multi-phenotypic integration despite residual immune-level differences. A distinct discordant configuration is shown in Panel C, where large barycenter separation co-occurs with intermediate-complexity, strongly asymmetric hull geometry dominated by the interaction component, capturing a mode of selective divergence in which phenotypic alignment is retained while survival and immune behaviors diverge. Finally, Panel D demonstrates that extreme geometric separation does not necessarily imply biological divergence: despite occupying distant regions of the latent space and forming high-complexity, moderately asymmetric polytopes, both components converge across survival, microenvironmental, and immune dimensions, yielding an Only Convergent circuitry. Together, Panels A–D delineate a coherent set of visually interpretable archetypes, showing how combinations of barycenter distance and polytope volume encode distinct modes of regulatory concordance, divergence, and convergence within the latent embedding.

### 3.6 Geometric interpretation and implications for regulatory phenotypes

Barycenter distance provides a direct numerical estimate of the geometric separation between the signature and interaction components of each circuitry. This geometric separation may coexist with functional alignment along specific biological axes. We refer to this pattern as separation-with-convergence, describing circuitries in which the sig and int components are spatially separated in latent space (large barycenter distance) yet exhibit concordant sign structure along specific annotated axes, including survival, microenvironmental, and immune dimensions. In such cases, barycenter distance captures latent-direction separation, whereas the concordance summary class reflects axis-specific alignment, allowing spatial separation to coexist with functional convergence in specific configurations, as illustrated in Figure 4D. In contrast, convex-hull volume quantifies the breadth of latent engagement, indexing how many phenotypic axes contribute to each side. The symmetry measure provides an additional layer of interpretability by identifying unequal contributions to latent complexity.

Together, these measures provide a unified quantitative description of regulatory architecture. Discordant circuitries represent divergent programs whose signature and interaction components occupy distinct regions of latent space. High-complexity geometries indicate multi-phenotype entanglement, whereas low-complexity geometries reflect narrowly tuned regulatory behavior. The predominance of discordant, high-complexity, asymmetric circuitries underscores the structural heterogeneity and phenotypic tension inherent to the regulatory systems captured in Dataset S1. These geometric findings provide a principled, quantitative foundation for subsequent biological analyses that examine how external annotations, such as metabolic pathways, distribute across the landscape defined here.

### 3.7 Distribution of metabolic superfamilies across geometric phenotypes

In the analyses that follow, stratification of metabolic superfamilies and pathways is interpreted primarily as a validation of the representational capacity and discriminatory resolution of the geometric framework, rather than as a definitive biological taxonomy of metabolic regulation.

#### 3.7.1 Stratification of metabolic superfamilies across barycenter-distance regimes

**FIGURE 5:**
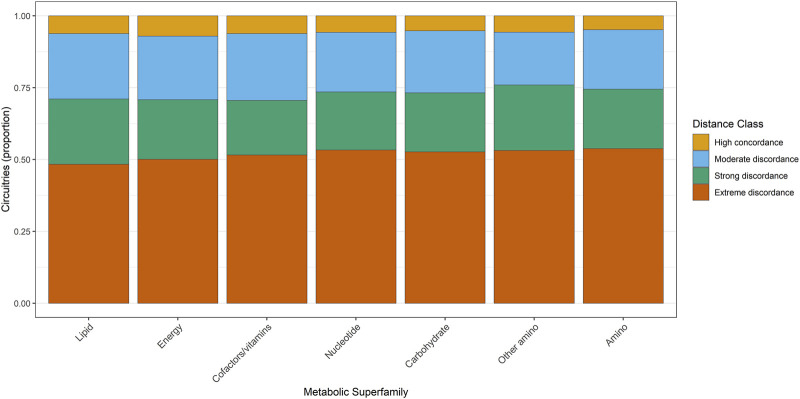
Proportional distribution of regulatory circuitries across geometric distance–implication classes and metabolic superfamilies. Bars represent the fraction of regulatory circuitries assigned to each of the four geometric distance–implication regimes within each metabolic superfamily. Metabolic superfamilies are defined according to KEGG classification and are ordered by Shannon entropy, such that superfamilies exhibiting broader dispersion across geometric regimes appear on the left. This entropy-based ordering highlights differences in geometric organization across metabolic domains: some superfamilies display highly concentrated geometric profiles, indicative of constrained or specialized regulatory behavior, whereas others exhibit more dispersed patterns, consistent with context-flexible or heterogeneous regulatory organization across the circuitry landscape.

The geometric discordance classification derived from barycenter distances revealed consistent large-scale stratification across metabolic superfamilies. All seven classes showed the full range of geometric regimes—high concordance, moderate discordance, strong discordance, and extreme discordance—but with markedly different internal proportions (Figure 5, Dataset S1).

Amino acid metabolism exhibited the most polarized distribution, with extreme discordance representing 53.8% of all circuitries assigned to this category (3,491 of 6,489), whereas high-concordance patterns constituted only 4.8% (313 circuitries). Carbohydrate metabolism followed a highly similar structure, with 52.7% of circuitries falling into extreme discordance and 5.2% into high concordance. Energy metabolism demonstrated a modest shift toward more concordant behavior, with 7.0% of circuitries in the high-concordance regime—the largest fraction among the superfamilies—yet the extreme-discordance class still dominated (48.0%).

Lipid metabolism and nucleotide metabolism displayed intermediate distributions, each maintaining ∼50% representation in the extreme-discordance regime (49.8% and 48.7%, respectively). Metabolism of cofactors and vitamins maintained the closest proportional balance between strong and extreme discordance (20.2% and 51.7%, respectively), but still showed only 6.2% high concordance. Across all metabolic classes, moderate and strong discordance each contributed approximately 20% of circuitries, indicating that most superfamilies occupy primarily discordant geometric regimes rather than uniformly concordant ones.

Collectively, these results demonstrate that geometric discordance is not uniformly distributed across metabolic superfamilies; rather, each superfamily exhibits a reproducible internal structure, with extreme discordance consistently dominating but with measurable differences in the magnitude of the high-concordance fraction.

**FIGURE 6:**
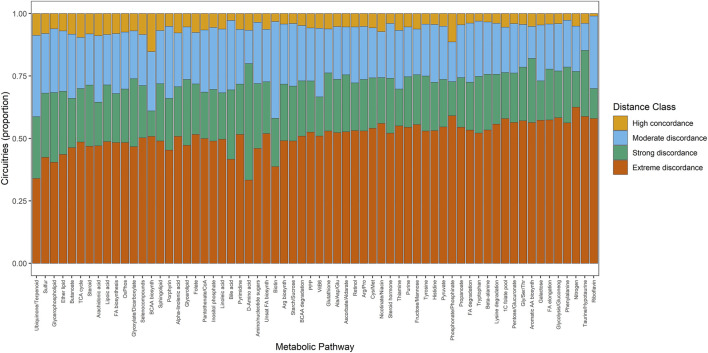
Proportional distribution of regulatory circuitries by geometric distance-implication classes across metabolic pathways. Each bar represents the proportion of circuitries assigned to one of four geometric distance regimes (High concordance, Moderate discordance, Strong discordance, Extreme discordance) within a given metabolic pathway. Pathways are ordered from left to right by Shannon entropy computed over the distance-class distribution, such that pathways exhibiting greater geometric pleiotropy (broad dispersion across discordance regimes) appear earlier. This representation highlights coherent gradients of geometric behavior across metabolic programs, distinguishing pathways that occupy highly constrained geometric states from those displaying structurally diverse circuitry configurations.

To resolve this organization at finer biological resolution, we next examined barycenter-distance regimes across individual metabolic pathways. Pathway-level stratification revealed coherent gradients of geometric behavior, ranging from pathways dominated by constrained, concordant configurations to those exhibiting broad dispersion across discordance regimes, indicative of pathway-specific regulatory divergence. This distribution is summarized in Figure 6, with pathways ordered by Shannon entropy to highlight differences in geometric pleiotropy.

#### 3.7.2 Geometric volume implications across metabolic superfamilies

**FIGURE 7:**
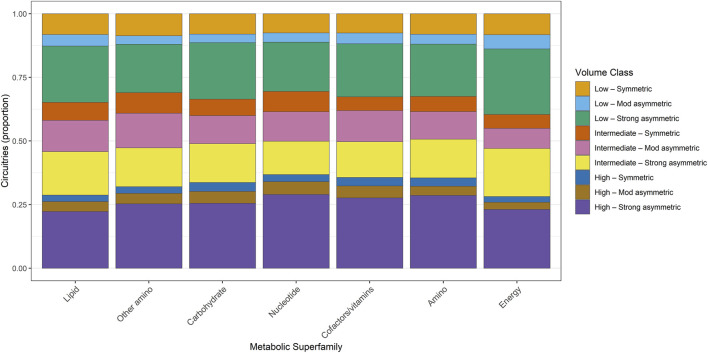
Proportional distribution of regulatory circuitries across geometric volume-implication classes for metabolic superfamilies. Each bar depicts the relative frequency of nine convex-hull–based geometric regimes, defined by latent dimensionality (Low, Intermediate, High) and symmetry/asymmetry of the circuitry volume. Entropy ordering positions superfamilies with high geometric complexity and broad multi-regime occupation to the left, and superfamilies with more restricted geometric signatures to the right. This visualization exposes systematic differences in latent geometric complexity across major metabolic domains.

Analysis of convex-hull implications revealed a heterogeneous distribution of latent-space complexity among metabolic superfamilies. All categories included circuitries spanning low-dimensional, intermediate-complexity, and high-complexity regimes, but the relative proportions differed substantially. These superfamily-level distributions of convex-hull–based geometric volume regimes are summarized in Figure 7 (Dataset S1).

Amino acid metabolism contained the highest absolute number of high-complexity geometry circuitries, with 44.3% falling into high-complexity multidimensional symmetric regimes and another 30.4% in high-complexity strongly asymmetric configurations. Low-dimensional flat geometries were comparatively rare (collectively 8.8%), indicating that this metabolic class is predominantly associated with higher-order latent deformation patterns.

Carbohydrate metabolism followed a near-identical pattern: 42.8% of circuitries aligned with high-complexity symmetric hulls, while 30.7% mapped to high-complexity strongly asymmetric geometries. Low-dimensional classes again represented <10% of the total. Lipid metabolism showed a similar hierarchical structure, with 43.7% of circuitries in high-complexity symmetric geometries and 28.7% in high-complexity strongly asymmetric geometries.

Energy metabolism exhibited a modestly higher proportion of low-dimensional flat geometries (12.2%) compared with other superfamilies, yet high-complexity regimes remained overwhelmingly dominant (symmetric: 40.3%, strongly asymmetric: 27.9%). Nucleotide metabolism and metabolism of cofactors and vitamins similarly exhibited high-complexity geometries as their prevailing configuration, each with >70% of circuitries occupying high-complexity symmetric or asymmetric regimes.

Taken together, these geometric volume analyses establish that most metabolic superfamilies are preferentially associated with complex latent geometries rather than low-dimensional configurations. Although the degree of asymmetry varies across categories, high-complexity symmetric hulls consistently emerge as the principal geometric phenotype across metabolic domains.

**FIGURE 8:**
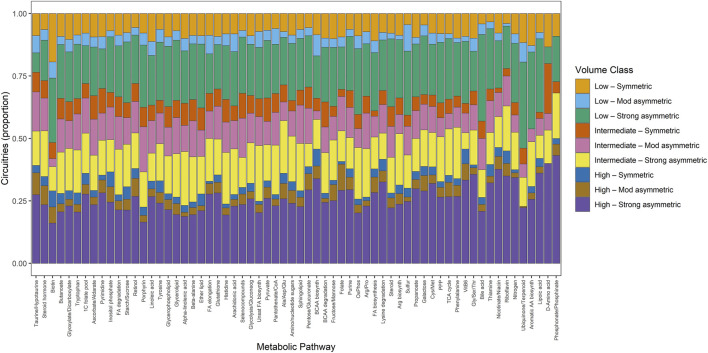
Proportional distribution of regulatory circuitries across geometric volume-implication classes for individual metabolic pathways. Bars represent the distribution across nine geometric volume regimes, capturing both latent dimensionality and convex-hull asymmetry. Pathways are ordered by Shannon entropy computed from the volume-class distribution, revealing a continuum from highly pleiotropic pathways that participate in diverse geometric architectures to specialized pathways concentrated in one or a few geometric regimes. The figure provides a fine-grained map of geometric heterogeneity across metabolic circuitry structures.

Extending this analysis to the pathway level revealed substantial heterogeneity in latent geometric complexity among individual metabolic programs. While some pathways were concentrated within a limited subset of volume-implication regimes, others spanned the full range of latent dimensionality and convex-hull asymmetry, consistent with pathway-specific multivariate engagement. This fine-grained distribution of volume-implication classes across metabolic pathways is shown in Figure 8, with entropy-based ordering emphasizing differences in geometric specialization versus pleiotropy.

A detailed stacked bar chart mapping the proportional distribution of molecular circuitries across individual, fine-grained Metabolic Pathways along the horizontal axis, analogous to Figure 6. The vertical axis measures circuitry proportions from 0 to 1. The bars are vertically segmented and color-coded according to the nine "Volume Class" categories seen in Figure 7. These segments represent combinations of polytope volume sizes ("Low", "Intermediate", "High") and their associated geometrical symmetries ("Symmetric", "Mod asymmetric", "Strong asymmetric"). The detailed plot reveals the specific spatial shape and size distribution of metabolic signatures across localized biological pathways.

### 3.8 Entropy-based quantification of geometric heterogeneity across concordance classes

We next examined how circuitries distribute across the full spectrum of multivariate concordance outcomes. By quantifying the proportion of circuitries assigned to each final concordance summary class and ordering these categories by Shannon entropy, we visualized the relative structural diversity underlying each outcome state (Supplementary Figure S4). The entropy-based ordering provides a neutral ranking that places classes with broader geometric dispersion earlier in the plot, facilitating comparison of distributional patterns across the entire concordance landscape. In this context, entropy captures the dispersion of circuitries across the four geometric regimes (high concordance, moderate discordance, strong discordance, and extreme discordance). Because these regimes encode distinct latent structural configurations of the underlying multi-omic circuitry, entropy provides a principled measure of whether biologically defined categories occupy a narrow versus diffuse region of the geometric space. Low entropy reflects structural concentration, indicating that circuitries within a concordance class converge toward a dominant geometric configuration. High entropy, in contrast, denotes a broad distribution across geometric regimes, implying that circuitries with similar biological outcomes may arise from widely varying multivariate architectures.

Applying this framework revealed a nontrivial decoupling between biological concordance and geometric organization. The classes Only Convergent and Only Divergent, which together represent the two largest categories in Dataset S1, exhibited markedly different entropy values despite their comparable sample sizes. Only Convergent displayed a higher entropy (∼1.30), indicating that these circuitries populate a broad range of geometric regimes without a single dominant configuration. This suggests that fully concordant multi-omic signals can emerge from diverse structural organizations, reflecting multiple mechanistic routes to geometric agreement. By contrast, only Divergent exhibited lower entropy (∼1.07), demonstrating a more concentrated geometric profile in which divergent biological signals disproportionately arise from a narrower subset of geometric configurations. This asymmetry implies that divergence in immune, phenotype, Cox, or survival dimensions may be driven by specific, structurally constrained geometric patterns, whereas full concordance is structurally more permissive.

Together, these findings demonstrate that entropy provides a sensitive discriminator of geometric heterogeneity across concordance categories and uncovers an emergent organizing principle: biological divergence tends to be geometrically constrained, whereas biological concordance is realized through a richer diversity of multivariate configurations. This insight strengthens the mechanistic interpretability of the geometric framework and supports the notion that distinct biological agreement states arise from characteristic, and quantifiable, geometric architectures.

### 3.9 Comparison with baseline discordance metrics

To assess whether the geometric discordance metric captures information beyond conventional vector-space discordance measures, we compared the barycenter distance metric (*dbary*, defined as the distance between signature and interaction barycenters in the shared PC1–PC3 embedding) with several baseline discordance measures derived from the same reconstructed 18-dimensional latent vectors (Dataset S2). These baseline measures included Euclidean distance in the latent space, cosine distance between vectors, survival-direction mismatch counts across endpoints (OS, DSS, DFI, and PFI), immune classification mismatch, tumor–normal direction mismatch, and absolute differences in microenvironment score. Barycenter distances recomputed from the reconstructed latent vectors closely matched the values reported in Dataset S1 (Spearman *ρ* = 0.998), confirming the reproducibility of the geometric computation. Correlation analysis showed that *dbary* was weakly associated with generic vector-space distance measures (Spearman *ρ* = 0.284 with Euclidean distance and *ρ* = 0.158 with cosine distance), indicating that the geometric metric is not reducible to simple vector-space distances. In contrast, *dbary* showed strong association with survival-direction discordance (Spearman *ρ* = 0.766), consistent with the structured contribution of survival axes within the latent representation. Correlations with simple categorical mismatches were minimal, including immune classification mismatch (Spearman *ρ =* 0.064) and tumor–normal direction mismatch (Spearman *ρ =* 0.028). These weak associations indicate that the geometric discordance metric is not reducible to categorical disagreement alone but instead reflects multidimensional divergence across integrated phenotypic axes.

To further assess the contribution of different axis families, we performed embedding robustness analyses in which specific axis groups were removed or the PCA preprocessing was modified. Removing survival axes substantially altered the geometric ranking (Spearman *ρ* = 0.046 relative to the reference embedding; regime agreement = 0.336), indicating that survival information constitutes a major contributor to the latent structure underlying barycenter separation. In contrast, removing the immune classification axis had minimal impact (Spearman *ρ* = 0.998; regime agreement = 0.965), suggesting that immune classification contributes comparatively little to barycenter separation in this representation.

An additional robustness check evaluated the effect of PCA scaling choices. When PCA was recomputed without variance scaling, the resulting barycenter-distance metric remained positively correlated with the main embedding (Spearman *ρ* = 0.214) and preserved approximately half of the discordance regime assignments (agreement = 0.51), indicating that the geometric discordance signal is not driven solely by variance standardization.

Together, these results indicate that the geometric discordance metric captures structured variation across multiple latent axes and is not reducible to conventional vector-space distance measures. The full correlation structure across baseline and geometric metrics is shown in Supplementary Figure S5.

To determine whether the observed distribution of concordance–discordance regimes could arise from random pairing between molecular signatures and regulatory interaction components, we evaluated the barycenter-distance structure using a permutation-based null model. Interaction embeddings were randomly reassigned within cancer-type strata, and the geometric embedding was recomputed for each permutation replicate (B = 500). Comparison with the resulting null distributions showed that the observed barycenter-distance structure deviates from expectations under random pairing. The observed mean barycenter distance exceeded the permutation-based null expectation (empirical p = 0.036). Circuitries classified as extreme discordance represented 51.2% of the dataset and were significantly enriched relative to the permutation-based null distribution (empirical p = 0.004). In contrast, highly concordant circuitries represented only 6.1% of the dataset and did not differ from the null expectation (empirical p = 0.776), indicating that the statistical enrichment is concentrated in the discordant tail of the barycenter-distance distribution. The corresponding null distributions are shown in Supplementary Figure S6, and detailed permutation statistics are provided in Dataset S2. These results provide statistical support for the interpretation of discordance regimes and demonstrate that the observed geometric organization cannot be explained by random pairing of regulatory and metabolic signatures.

### 3.10 Shiny-based interpretation of geometric signatures

Interactive exploration of OncoMetabolism regulatory circuitry geometry was performed using the *SigPolytope Shiny* application, which enables direct visualization and comparison of convex-polytope representations of multi-omic regulatory circuitries. This application provides a practical interpretive interface for the geometric structures described in this atlas. Through navigation of the shared latent space, users can examine how circuitries distribute across regimes of concordance and discordance, levels of geometric complexity, and degrees of asymmetry between signature and interaction components.

Joint visualization of dual polytopes together with automatically generated textual summaries allows abstract geometric properties—such as barycenter displacement and multidimensional expansion—to be systematically linked to biological and clinical attributes, including survival associations, tumor microenvironmental context, and immune phenotypes. This interactive framework supports the identification of representative circuitries, comparison of structurally related signatures, and detection of regulatory divergences that are not readily apparent from conventional vector-based or network-based representations.

## 4 Discussion

### 4.1 Rethinking how multi-omic regulatory interactions are represented

This study is methodological and conceptual in scope: it introduces a geometric framework for representing and comparing multi-omic signatures and regulatory circuitries, rather than extending or replacing existing biological atlases or making direct causal claims about specific molecular mechanisms. The multidimensional structure revealed through this framework necessitates a fundamental reconsideration of how multi-omic interactions are represented, visualized, and interpreted. Multi-omic circuitries are not linear associations; they are high-dimensional informational entities whose behavior arises from coordinated contributions across molecular, phenotypic, immune, microenvironmental, and clinical axes. Yet the dominant visual paradigms employed in the field—network maps, alluvial flows, layered cubes, and force-directed 2D or 3D graphs—compress this complex architecture into schematic representations that obscure structural features essential for biological interpretation. The analyses presented here illustrate that such representational flattening is more than an aesthetic constraint: it may act as a conceptual bottleneck that limits what can be inferred from multi-omic data.

At the level of latent representation, the geometric framework introduced here should be understood as complementary to existing embedding and representation-learning approaches rather than as a replacement for them. The geometric framework presented here is intended as a representational and analytical formalism for organizing multi-omic regulatory circuitries rather than as a predictive modeling system. The geometric descriptors derived from the latent representation—such as barycenter displacement and convex-hull geometry—provide structural measures that enable principled comparison of circuitries across cancer contexts. While these properties suggest potential applications in areas such as biomarker redundancy analysis, prioritization of regulatory programs, or hypothesis-driven exploration of immune and metabolic phenotypes, the present study does not attempt to evaluate predictive performance or clinical decision tasks. Instead, the framework establishes a structured geometric representation that can support future studies aimed at testing such applications in dedicated predictive or experimental settings.

Dimensionality-reduction techniques such as principal component analysis, manifold learning, diffusion maps, or graph-based embeddings provide coordinate systems that enable high-dimensional biological data to be visualized and compared within a shared latent space. The SigPolytope framework can therefore be interpreted in relation to these representation-learning approaches. In most embedding frameworks, biological entities are represented as points within a latent coordinate system derived from high-dimensional measurements. In contrast, the present framework treats each regulatory circuitry not as a single point but as a geometric object whose internal structure can be analyzed through convex-hull geometry. The objective is therefore not to replace embedding methods but to extend them by introducing object-level structural descriptors—such as hull volume, anisotropy, and barycenter separation—that quantify the multidimensional organization of regulatory programs within the latent space. In this sense, SigPolytope can be viewed as a geometric layer operating on top of conventional latent embeddings rather than as an alternative embedding algorithm.

In the present framework, PCA serves this role by establishing a shared coordinate system in which regulatory circuitries are co-embedded. The conceptual contribution of the SigPolytope approach lies in extending this representation beyond point-based embeddings. Instead of representing each circuitry as a single vector in latent space, the framework encodes circuitries as convex geometric objects whose structural properties can be analyzed through measures such as barycenter displacement and convex-hull geometry. This object-based representation allows regulatory configurations to be compared through geometric relationships that are difficult to summarize using pairwise similarity measures between vectors alone, thereby providing a complementary perspective on the organization of multi-omic regulatory systems.

Because the framework relies on PCA embedding of heterogeneous latent variables, it is important to note that PCA represents one possible coordinate system for organizing the latent representation. In the present study, PCA was selected because it provides a deterministic and interpretable linear embedding that allows regulatory circuitries to be projected into a shared coordinate system suitable for atlas-scale geometric comparison. However, alternative embedding strategies could be explored in future implementations of the framework. For example, nonlinear manifold methods or distance-preserving embeddings could provide complementary representations of the latent structure when relationships among latent variables depart from linear assumptions. Such approaches may offer additional flexibility for capturing complex geometric relationships in latent space, although their suitability would need to be evaluated with respect to interpretability, stability, and cross-dataset comparability.

Within this conceptual framework, the geometric framework does not replace conventional vector-distance metrics but complements them by summarizing the structural organization of regulatory configurations in latent space. Whereas standard distance measures quantify pairwise similarity between vectors, the geometric representation captures how the combined phenotypic, survival, and microenvironmental dimensions are jointly organized around the circuitry signature. This provides added value beyond standard distance measures by summarizing the multidimensional configuration of regulatory relationships rather than only pairwise similarity. Consistent with this interpretation, benchmarking analyses comparing the barycenter-distance metric with conventional vector-space distances and categorical mismatch measures show only weak monotonic association, indicating that the geometric descriptors summarize multiaxial configuration patterns that are not reducible to standard distance statistics.

Within this geometric representation, convex-hull volume provides an informative descriptor of how broadly a regulatory circuitry engages the phenotypic axes encoded in the latent representation. In the SigPolytope framework, these axes correspond to integrated biological signals including tumor–normal behavior, survival associations across four survival endpoints (OS, DSS, DFI, and PFI), immune classification, microenvironmental context, and correlation structure between molecular components. Differences in hull volume therefore reflect differences in phenotypic dimensionality of regulatory influence: circuitries dominated by a small number of phenotypic axes produce compact geometries, whereas circuitries whose regulation simultaneously affects multiple phenotypic layers generate larger multidimensional expansions. In this sense, convex-hull volume serves as a proxy for the breadth of phenotypic integration encoded by the circuitry, rather than representing empirical dispersion of individual molecular measurements.

Network diagrams, whether in two or three dimensions, excel at depicting connectivity but lack any meaningful metric geometry. Physical proximity between nodes reflects the behavior of a layout algorithm rather than biological organization. Circuitries that diverge strongly in latent space may appear arbitrarily close if the force-directed algorithm attracts them, while highly similar entities may be placed far apart for reasons unrelated to biology. Networks, therefore, encode who connects to whom, but cannot express how regulatory signals distribute across latent dimensions, nor the structural tensions, concordances, or divergences that emerge from multi-omic integration.

Alluvial diagrams and multi-omic cubes provide alternate perspectives yet remain fundamentally constrained. Alluvial representations capture continuity—how features migrate across categories or pathway memberships—but they lack any ability to depict curvature, anisotropy, or latent-dimensional deformation. Multi-omic cubes restore interpretability along observable axes such as tumor–normal directionality, immune polarization, or survival tendencies, but their fixed coordinate system cannot encode the internal covariance structure that governs circuitry geometry. They cannot reveal asymmetric expansion, multidirectional deformation, or barycenter displacement—structural elements that are central to understanding phenotypic divergence.

The convex-polytope framework introduced here directly addresses these limitations. By embedding each circuitry as a pair of 18-dimensional latent regulatory vectors, expanding them into barycenter-centered point clouds, and projecting them into a shared geometric space, the resulting dual hulls preserve structural information rather than abstracting it away. Hull volume quantifies latent complexity, barycenter distance quantifies discordance, and anisotropy captures directional dominance across phenotypic or mechanistic axes. These properties derive from the latent vectors constructed from the integrated omic and phenotypic features rather than from visualization heuristics or graph-layout algorithms.

This distinction becomes decisive when examining the Dataset S1. More than half of all circuitries fall into extreme-discordance regimes, and more than 70% occupy high-complexity symmetric or strongly asymmetric geometries. No alluvial diagram, cube, or network graph can readily reveal this structural prevalence, nor explain why discordant, high-dimensional asymmetric circuitries dominate metabolic regulatory space. Most directly, the dual-polytope geometry exposes these large-scale organizational principles: how signature and interaction components diverge across latent dimensions, how multi-axis deformation corresponds to heterogeneous configurations within the latent representation, and how regulatory modules distribute systematically across metabolic programs.

Accordingly, geometry is not simply an alternative visualization; it constitutes a representational framework that retains multidimensional structure and enables interpretive statements unavailable through conventional approaches. The convex hull does not merely depict a circuitry—it is the circuitry, expressed as a measurable geometric object whose properties summarize structural relationships encoded in the latent representation rather than imposed graphical conventions. This geometric framing anchors the remainder of the Discussion, offering a rigorous foundation for interpreting phenotypic discordance, latent complexity, and the distribution of metabolic processes across geometric regimes.

Because a hull is defined over a set of points in latent space, it admits intrinsic measurements that translate naturally into biological interpretation. Hull volume reflects multidimensional amplitude, indexing the breadth of phenotypic and regulatory dispersion. Principal-axis orientation, derived from the covariance structure of latent coordinates, reveals which biological dimensions dominate a circuitry’s behavior—whether regulation is driven by survival, immune directionality, tumor–normal polarity, or microenvironmental influence. Curvature and anisotropy differentiate coherent single-axis programs from modular, multi-axis, or branching architectures.

These geometric metrics formalize patterns that biologists often perceive qualitatively but cannot quantify using list-based or vector-based representations, thereby providing a robust bridge between biological intuition and computational structure. Importantly, geometric formalism does not replace biological reasoning; it strengthens it by making structural relationships explicit. A convex hull can show that a seemingly unified signature is in fact topologically fragmented, prompting biological or regulatory subdivision. Conversely, it may reveal that two signatures with distinct molecular compositions are geometrically superimposable, implying functional equivalence and potential biomarker redundancy (Figure 4B, F4_HTML; circuitry_id = “LGG-6708/LGG-5904”).

Through this lens, core biological concepts—heterogeneity, redundancy, convergence, divergence, modularity, dominance—cease to be metaphors and become quantifiable geometric properties. By applying geometric reasoning to multi-omic signatures, analysis acquires explanatory power not possible with traditional representations. The convex hull thus becomes not merely a visualization artifact, but an epistemological bridge linking multi-omic evidence to biological meaning.

### 4.2 Geometric embedding of cancer as a multidimensional system

Recent refinement of the hallmark framework has articulated cancer as a system organized along four conceptual dimensions—acquired functional capabilities, hallmark-enabling phenotypic characteristics, hallmark-conveying cells of the tumor microenvironment, and systemic interactions—intended to distill mechanistic complexity into orthogonal explanatory axes (Hanahan, 2026).

Although these dimensions were formulated qualitatively, they align naturally with the type of multidimensional structure captured by the geometric representation of omic signatures developed here. In the present framework, each regulatory circuitry is embedded in an 18-dimensional latent space integrating regulatory, phenotypic, immune, microenvironmental, and clinical attributes, and is represented as a convex polytope whose shape and position summarize its internal organization.

From this perspective, functional capabilities are reflected in dominant directions of variance associated with survival effects, tumor–normal shifts, and pathway engagement; enabling phenotypic characteristics are reflected in dispersion and anisotropy across latent axes capturing genomic, regulatory, and stress-response constraints; hallmark-conveying microenvironmental cells and immune states contribute structured deformation along axes encoding tumor microenvironment and immune contexture; and systemic interactions are indirectly represented through survival endpoints and global phenotypic gradients. Importantly, this correspondence does not imply a one-to-one mapping between specific hallmarks and latent coordinates. The reference to the hallmarks framework should therefore be interpreted as a conceptual analogy highlighting the multidimensional organization of cancer biology rather than as a formal statistical mapping between geometric descriptors and individual hallmark categories. Instead, it indicates that the conceptual dimensions proposed for cancer biology emerge empirically as coupled geometric degrees of freedom when real multi-omic data are embedded in a shared latent space. In this sense, polytope projections provide a quantitative instantiation of multidimensional cancer organization, enabling the qualitative framework of the four dimensions to be interpreted alongside measurable geometric properties such as distance, volume, and anisotropy, while also exposing regimes of discordance and structural heterogeneity not accessible to conceptual models alone.

### 4.3 Geometry as a foundation for signature comparison, classification, and translational relevance

The geometric framework introduced above reorients the analysis of multi-omic signatures from describing each signature in isolation to understanding how signatures relate to one another as structured informational entities. Once signatures are embedded in a common latent coordinate system and expressed as convex polytopes, similarity, divergence, redundancy, complementarity, and mechanistic distance become measurable quantities rather than qualitative impressions. Geometry thus shifts the interpretive focus from enumeration to comparison and from annotation to structure.

Two signatures that occupy overlapping hull volumes can be interpreted as functionally aligned within the latent representation—even if they share few or no molecular components—because geometric overlap reflects concordant behavior across the 18 latent axes that encode correlation structure, tumor–normal shifts, survival tendencies, microenvironmental influence, and immune state (Figure 4). Conversely, signatures that diverge markedly in orientation, volume, or curvature occupy distinct regions of latent space and therefore encode distinct biological programs (Figure 3). Through this lens, geometric representation transforms signature analysis from a descriptive exercise into a comparative science of multidimensional biological architectures.

Because geometric relationships encode concordance, divergence, and redundancy across clinically relevant latent dimensions, this comparative capacity has immediate translational consequences. In precision oncology, numerous candidate signatures are often proposed for the same phenotype, pathway, or clinical endpoint. Lacking a structural framework, these signatures are typically treated as independent entities, leaving redundancy, equivalence, or complementarity to conjecture rather than demonstration. The convex-polytope representation resolves this ambiguity: redundancy appears as volumetric overlap, equivalence as geometric congruence, and complementarity as divergence along orthogonal axes. Geometry, therefore, enables principled prioritization—redundant signatures can be collapsed, divergent signatures can be stratified, and only the most structurally informative candidates should advance to clinical validation.

The interpretive power of geometric reasoning becomes particularly clear in immuno-oncology, where immune states define well-characterized but multidimensionally coupled phenotypic regimes. Immune-active signatures that converge on similar latent phenotypes produce hulls that occupy overlapping regions of geometric space (Figure 4), while immune-excluded or immune-suppressed signatures diverge along axes associated with stromal shielding, metabolic reprogramming, or immune evasion (Figure 3). This stratification is not imposed; it emerges directly from the internal geometry of each circuitry. In this context, geometry becomes a functional classifier: hot-aligned geometries identify candidate predictors of immunotherapy benefit, whereas cold-aligned geometries reveal signatures associated with immune exclusion, immunoediting trajectories, or therapeutic resistance.

Beyond immune phenotypes, geometric divergence offers a rational framework for therapeutic inference. Candidate drugs, synthetic-lethality hypotheses, and combination strategies can be prioritized based on geometric distance in the latent regulatory representation—defined geometrically—rather than superficial gene overlap or heuristic similarity scores. Signatures separated by large barycenter distances represent structurally discordant regulatory programs in latent space that may respond to orthogonal therapeutic pressures. Signatures with minimal barycenter separation and congruent volumes, by contrast, highlight biological redundancies that should be avoided when constructing multi-signature panels.

More broadly, geometric representation provides a foundation for de-redundancy, benchmarking, and cohort stratification across oncology applications. Two prognostic or predictive signatures purported to stratify the same patient population can be compared directly: if their hulls are congruent, only one is necessary; if they diverge, each captures orthogonal axes of tumor biology, justifying complementary use. Geometry thus transforms signatures from narrative artifacts into quantitative biomarker objects—entities that can be ranked, merged, or discarded based on structural justification rather than interpretive intuition.

Beyond the regulatory circuitry atlas examined in the present study, the geometric framework may support a range of comparative analyses of molecular signatures across diverse biological contexts. Because the representation treats each signature as a geometric object embedded in a shared latent coordinate system, convex-polytope geometry provides a structured basis for evaluating redundancy, divergence, or complementarity among candidate biomarker panels. This representation may also facilitate cross-cohort harmonization of multi-omic signatures generated from independent studies, enabling signatures derived in different datasets to be compared within a common latent framework. In such settings, geometric descriptors offer a principled way to summarize the multidimensional configuration of regulatory programs rather than relying exclusively on pairwise similarity between vectors. Evaluation of these potential applications will require dedicated studies designed to assess their analytical and translational utility.

Although barycenter distance and hull-volume ratio jointly contribute to discordance classification, these metrics quantify orthogonal aspects of geometry: positional displacement versus latent-dimensional expansion. Their combination accounts for the broad nonlinear behavior observed in extreme-discordance circuitries and underscores the multidimensional nature of regulatory divergence. By giving signatures a geometry, this framework provides a rigorous foundation for comparative reasoning, biologically meaningful classification, and translational prioritization. Geometry distinguishes signal from redundancy, mechanism from noise, and biological architecture from statistical coincidence. It is not an embellishment of visualization but an enabling condition for the next-generation of mechanistically grounded, clinically credible biomarker science.

### 4.4 Limitations and caveats

Although the geometric framework presented here provides a principled method for representing, comparing, and interpreting multi-omic regulatory circuitries, several limitations should be acknowledged. These limitations do not undermine the framework’s core contributions but clarify the boundaries within which the current implementation should be interpreted and extended.

First, the latent space is constructed from an 18-dimensional tensor that captures the dominant measurable axes of circuitry behavior—correlation structure, tumor–normal directionality, survival tendencies, microenvironmental gradients, and immune states. While these variables encompass the major phenotypic dimensions encoded in the OncoMetabolismGPS atlas, they are not exhaustive. Importantly, the conclusions derived here depend on relative geometric organization rather than exhaustive axis coverage. Additional regulatory layers, such as chromatin topology, spatial transcriptomics, metabolic flux measurements, or treatment-induced perturbations, may introduce new axes of variation not captured by the present latent construction. Future versions of the atlas will benefit from integrating these modalities, particularly in contexts where regulatory behavior is governed by non-transcriptomic constraints.

Second, the PCA projection used to obtain a common geometric coordinate system necessarily compresses high-dimensional variation into three dimensions for visualization. This projection preserves dominant variance but cannot represent all latent interactions with equal fidelity. While the convex-polytope operations occur in the full 18-dimensional space before projection, the interpretability of hull shape, curvature, or anisotropy may still be influenced by the geometry of dimensionality reduction. Alternative embeddings (e.g., manifold learning, diffusion geometry, generalized linear factor models) may reveal orthogonal structures not captured by PCA and warrant comparison in future studies. Although the numerical thresholds used to define barycenter-distance regimes are heuristic, they are applied uniformly across all circuitries and yield stable qualitative patterns across cancer types, metabolic classes, and regulatory contexts. These thresholds serve as operational boundaries for summarizing continuous geometric variation rather than as sharp biological cutoffs, and the resulting regime structure is robust to moderate threshold perturbation.

Third, the convex-hull expansion relies on symmetric perturbations around each latent coordinate, allowing hulls to encode multidimensional deformation around a barycenter. Although this perturbation strategy preserves numerical stability and structural interpretability, it represents one of many possible ways to induce local geometric neighborhoods while preserving strict comparability across thousands of circuitries analyzed under identical geometric constraints. More complex or data-adaptive expansions—such as anisotropic perturbation kernels, distribution-based vertex generation, or probabilistic hulls—may refine the geometric representation in settings where circuitry behavior is highly non-linear.

Fourth, the interpretations presented here depend on the accuracy and completeness of underlying omic measurements. Batch effects, missingness patterns, or biases in clinical annotation can influence latent-axis contributions, especially in phenotypes with sparse or noisy endpoints. Although the imputation, normalization, and quality-control steps employed in the OncoMetabolismGPS pipeline adhere to stringent standards, residual artifacts may propagate into latent geometry. Cross-cohort validation, perturbation testing, and sensitivity analyses will be essential for establishing the robustness of the geometric classes across datasets, disease contexts, and platforms.

Fifth, the framework models each circuitry as a static entity derived from bulk omic measurements. This limitation reflects data availability rather than a constraint of the geometric formalism itself. Tumors, however, evolve dynamically, and single-cell heterogeneity, clonal substructure, and microenvironmental flux may generate temporal or spatial geometry shifts. Extending the convex-polytope model to longitudinal datasets or to per-cell circuitry embeddings may reveal regulatory dynamics that remain inaccessible in static representations.

Finally, although geometry captures structural relationships with high fidelity, it does not encode causal directionality. Barycenter displacement, hull inflation, and axis alignment quantify how signatures relate in latent space but do not specify why these relationships arise or which regulatory interactions drive them. Integrating geometric representations with causal graph inference, perturbation experiments, or mechanistic pathway models will be an important next step toward linking structure with mechanistic explanation.

Collectively, these limitations underscore the need for continued methodological refinement while reinforcing the conceptual strength of the geometric approach. The convex-polytope framework establishes a scalable foundation for representing multi-omic regulatory entities, and its limitations highlight natural directions for extending the model into causal, dynamic, and multimodal domains.

### 4.5 Concluding remarks

This work reconceptualizes omic signatures as multidimensional informational entities whose structure and biological meaning are inherently geometric, rather than vectorial or list-based. By embedding 24,796 metabolic regulatory circuitries into an 18-dimensional latent space and representing each as a dual convex polytope, we establish that signatures possess definable geometric identities—comprising barycenter displacement, latent complexity, anisotropy, and volume asymmetry—that capture structural relationships among phenotypic and regulatory dimensions that are difficult to represent using conventional approaches.

A key insight from the atlas is that discordance, not concordance, dominates the regulatory landscape. Most circuitries fall into strong or extreme discordance regimes and exhibit high-dimensional, frequently asymmetric geometries. These findings overturn the implicit assumption that multi-omic signatures function as coherent, unified objects; instead, they behave as structurally heterogeneous constructs whose biological meaning emerges from multi-axis deformation patterns rather than from molecular membership alone.

By treating each circuitry as a measurable geometric object, this framework enables principled comparison, stratification, and prioritization of signatures. Overlapping hulls indicate structural similarity in the latent representation independent of shared molecular components; divergent hulls quantify geometric separation between regulatory profiles; and symmetric or asymmetric volumes distinguish balanced from imbalanced phenotypic engagement. These geometric relationships provide a structural framework for comparing regulatory signatures, which may assist exploratory analyses such as biomarker de-redundancy assessment and interpretation of immune-associated phenotypic patterns.

More broadly, geometric formalism bridges biological intuition with computational structure. Properties traditionally described qualitatively—heterogeneity, modularity, convergence, divergence, dominance—become computable traits embedded in latent space. This transition from narrative to structure defines the conceptual pivot of the present work and establishes geometry as a rigorous language for expressing the organizational principles of multi-omic regulatory systems.

The resulting geometric atlas provides a scalable foundation for future multi-omic discovery. As emerging data modalities—including spatial transcriptomics, chromatin topology, metabolic flux profiling, and longitudinal tumor dynamics—are integrated into latent space, the polytope representation will expand to capture increasingly complex regulatory architectures. This trajectory points toward a new analytic paradigm in which mechanisms, phenotypes, and contexts are represented as geometric objects amenable to comparison, classification, and causal inference.

By giving omic signatures a geometry, we equip multi-omic science with a structural vocabulary capable of capturing the richness, heterogeneity, and emergent organization of cancer biology.

Future applications may include comparative analysis of biomarker redundancy and the exploration of how geometric representations could support cross-cohort harmonization of signatures across independent datasets.

The geometric framework and atlas presented here constitute not only an analytical resource but a conceptual foundation for building the next-generation of mechanistically grounded, clinically actionable biomarker systems.

## Data Availability

All data and R source code generated or analyzed during this study, including processed multi-omic matrices, derived geometric features, and annotated regulatory circuitry tables, are provided in the Supplementary Information and associated Supplementary Tables (Supplementary Source Code S1 and S2). The complete computational codebase used to construct the latent representations, geometric embeddings, and figures is publicly available via the SigPolytope repository (https://github.com/BioCancerInformatics/SigPolytope), together with interactive HTML figures used during analysis and quality control. Static, publication-grade figures shown in the manuscript were regenerated deterministically from the underlying data using non-interactive plotting pipelines. Interactive web resources for exploring the symbolic representation of multi-omic signatures and their associated regulatory circuitries, originally introduced in our previous work (11), remain publicly accessible through the CancerRCDShiny browser (https://cancerrcdshiny.shinyapps.io/cancerrcdshiny/) and the Multi-omic OncometabolismGPS Shiny platform (https://oncometabolismgps.shinyapps.io/Multi-omicOncometabolismGPSShiny/). These tools allow users to inspect the layered associations that compose each signature and to examine regulatory circuitry organization across biological, phenotypic, and clinical dimensions.
