# Supplementary figures and images for: Geometric multidimensional representation of omic signatures
Source: Front Bioinform. 2026 Apr 17;6:1806975. doi: 10.3389/fbinf.2026.1806975 (PMC13133092; doi:10.3389/fbinf.2026.1806975)

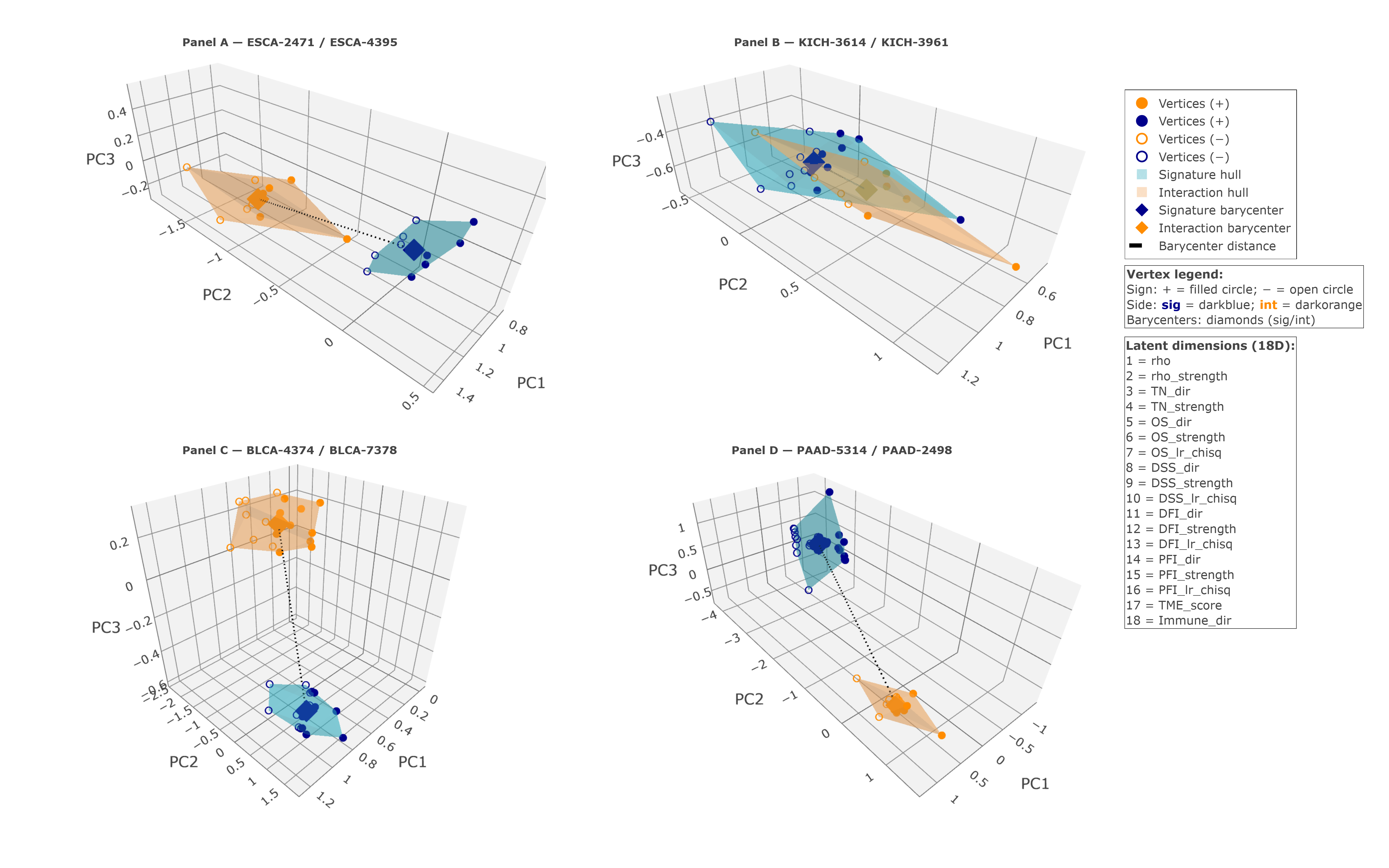

Supplement: Supplementary file 1 [file Image3.tiff]

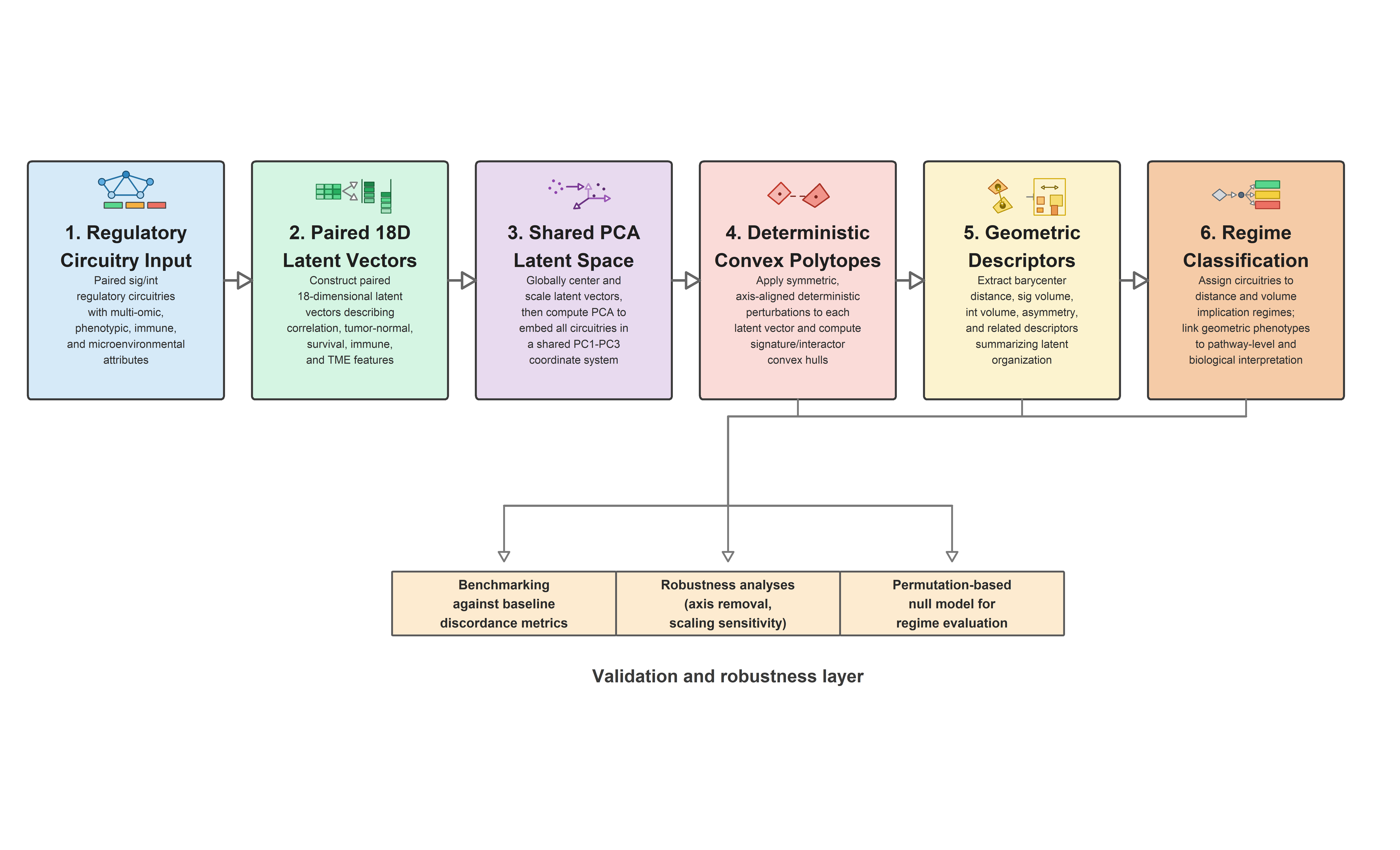

Supplement: Supplementary file 2 [file Image1.tiff]

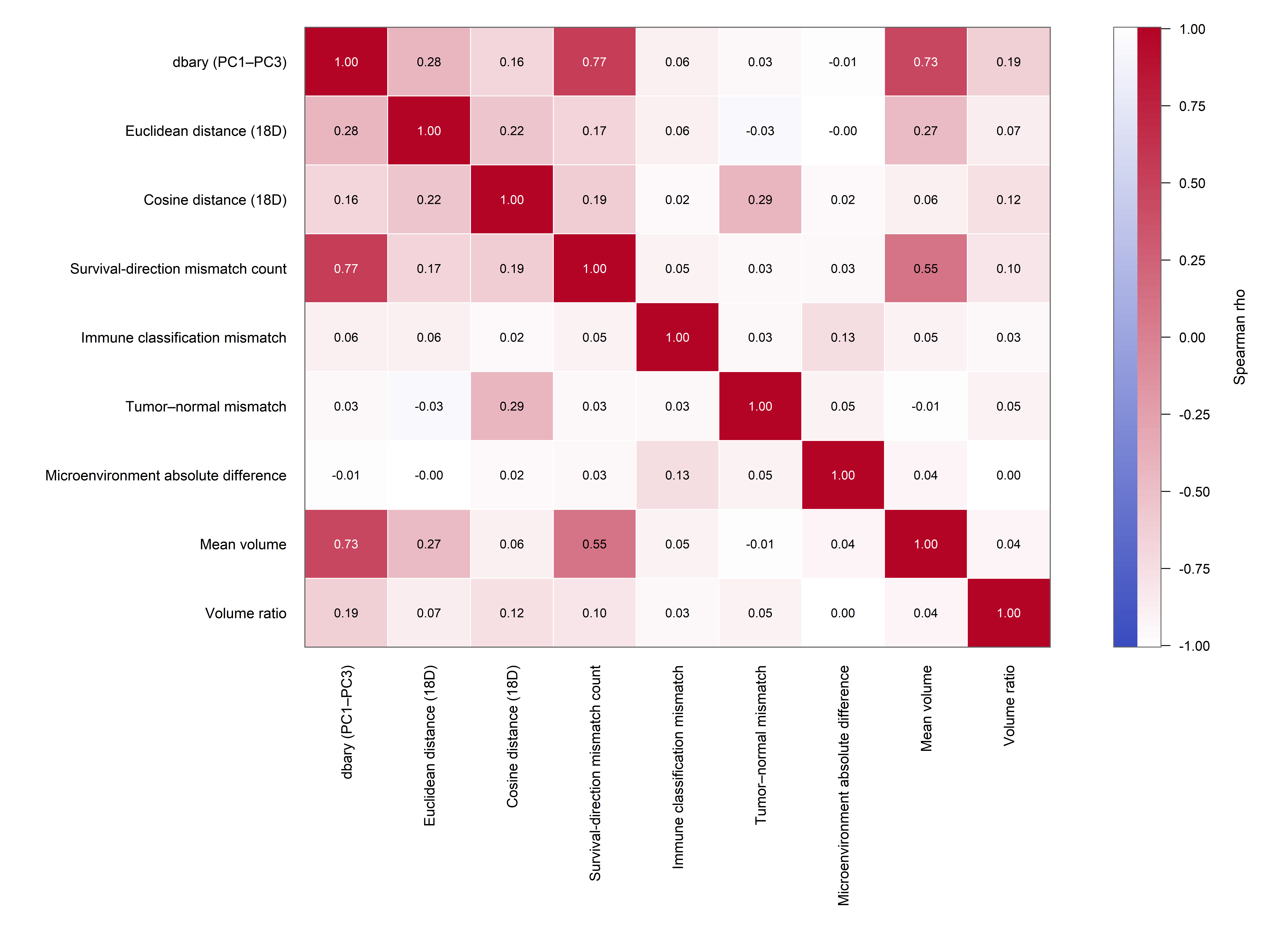

Supplement: Supplementary file 5 [file Image5.tiff]

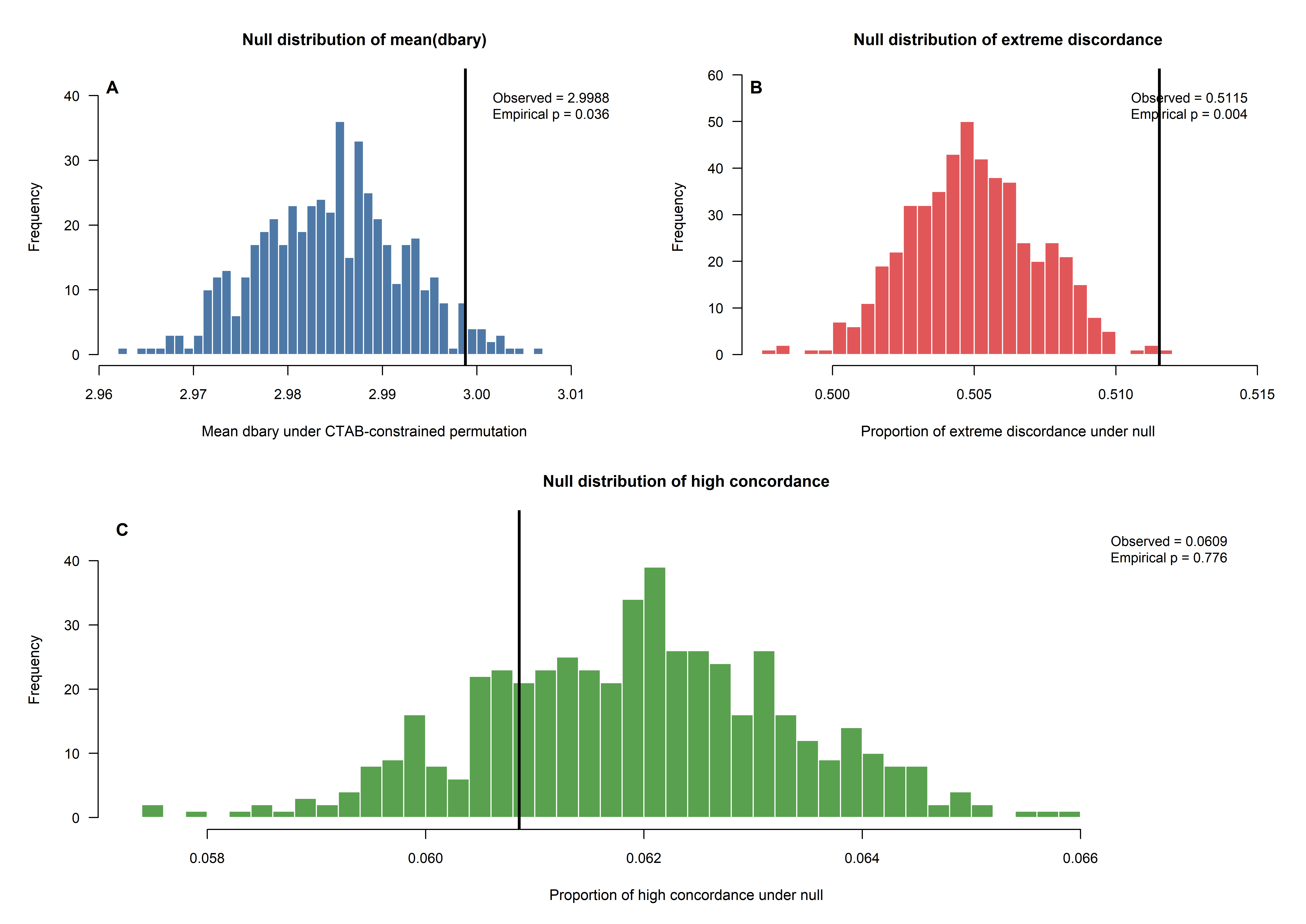

Supplement: Supplementary file 7 [file Image6.tiff]

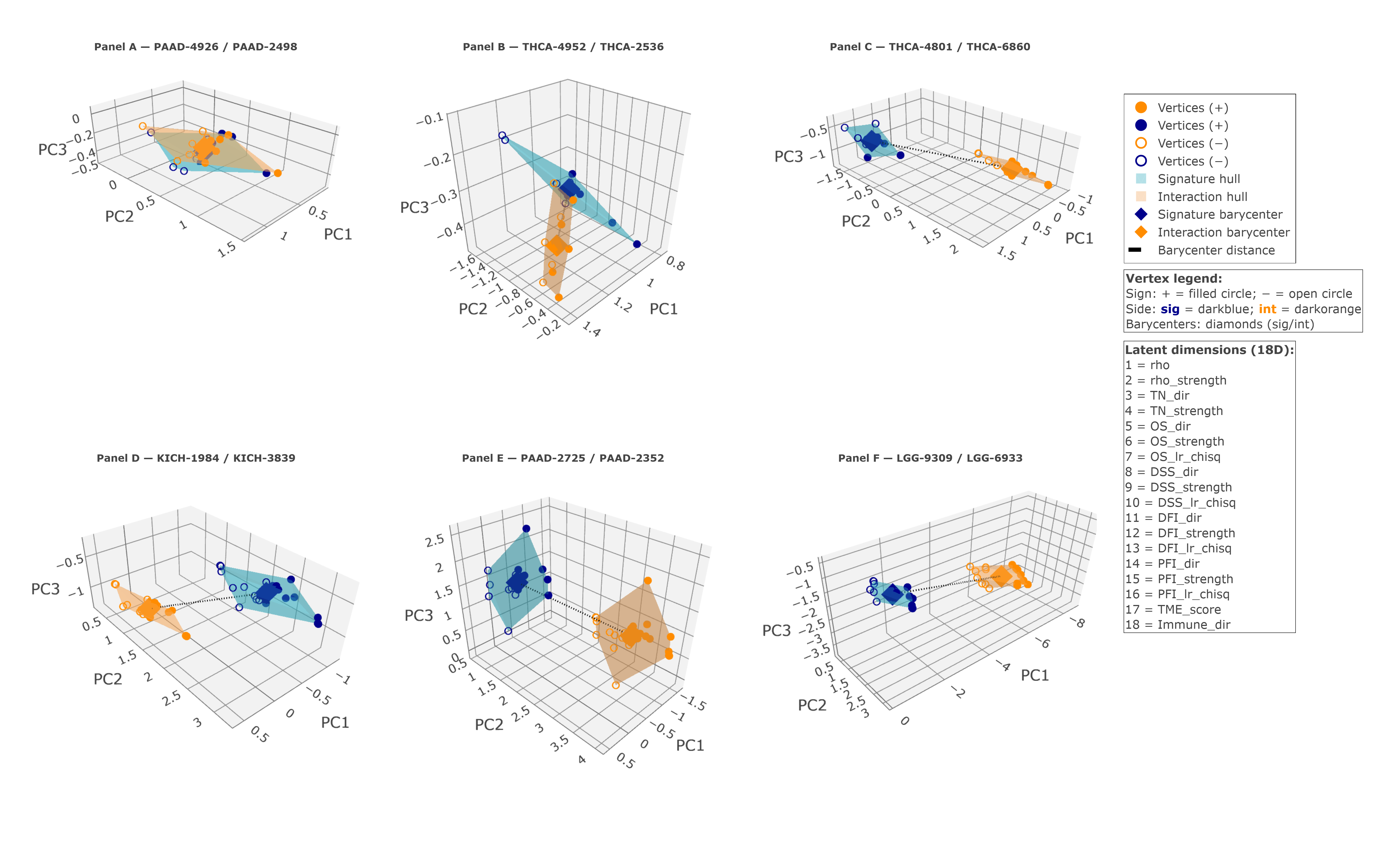

Supplement: Supplementary file 8 [file Image2.tiff]

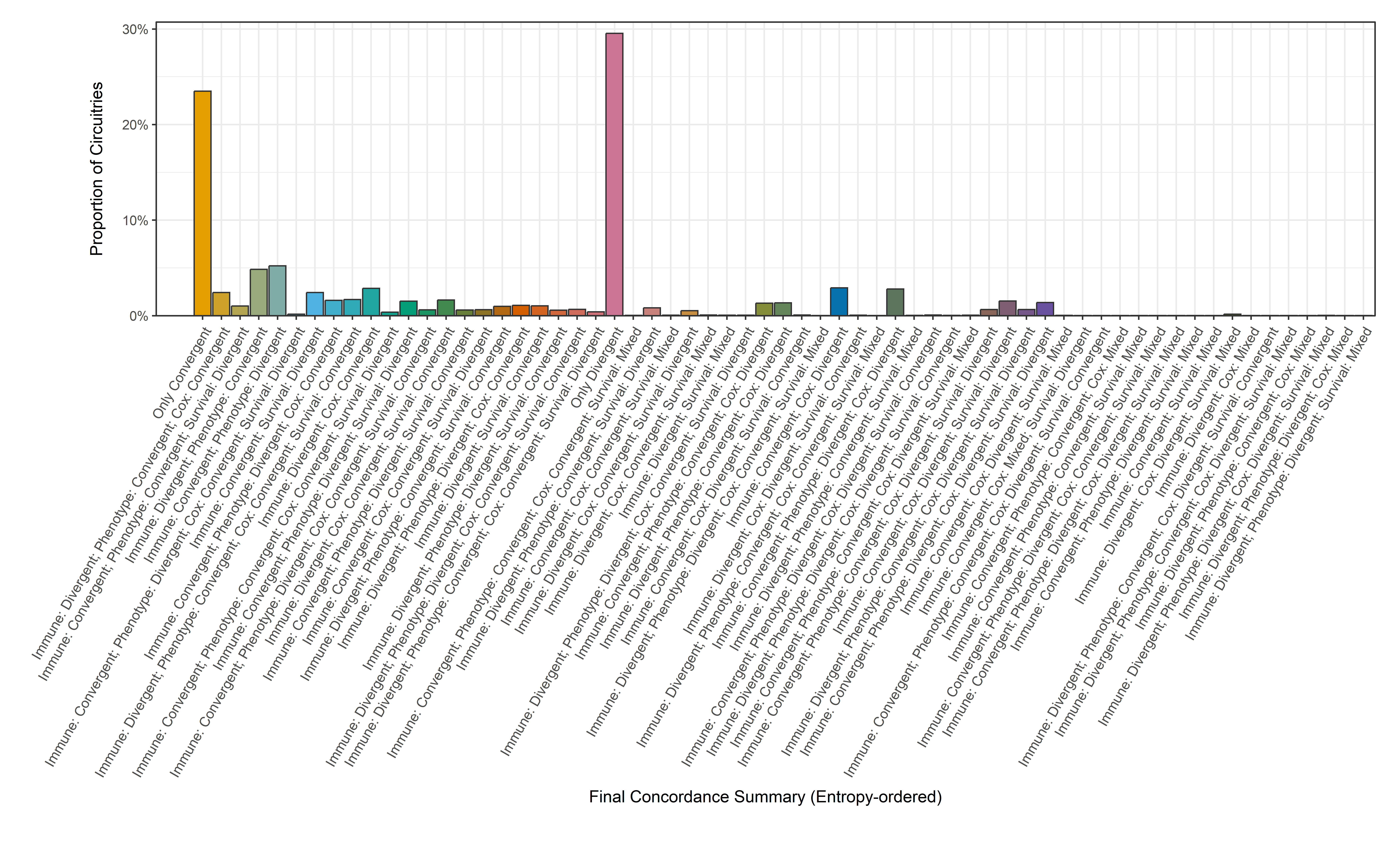

Supplement: Supplementary file 9 [file Image4.tiff]
